# Prevalence of *Mollicutes* in pregnant women undergoing high-risk prenatal care at a maternal and child reference unit in Bahia, Brazil

**DOI:** 10.1017/S0950268825100137

**Published:** 2025-06-25

**Authors:** Fabrícia Almeida Fernandes Santana, Jéssica Bomfim, Mariana Ferraz, Victória Cardoso, Jassy Borges, Danielle Souto de Medeiros, Maurício Grijó, Guilherme B. Campos, Lucas Miranda Marques

**Affiliations:** 1 Microbiology Department, State University of Santa Cruz (UESC), Ilhéus, Brazil; 2 Biointeraction Department, Multidisciplinary Institute of Health, Federal University of Bahia (UFBA), Vitória da Conquista, Brazil

**Keywords:** abortion, *Mollicutes*, mycoplasmas, ureaplasma, high risk prenatal, pregnancy

## Abstract

During pregnancy, colonization by genital mycoplasmas may be associated with adverse outcomes. This study was conducted to investigate the prevalence of four species of *Mollicutes* (*Mycoplasma hominis*, *Mycoplasma genitalium*, *Ureaplasma parvum*, and *Ureaplasma urealyticum*) in pregnant women receiving high-risk prenatal care and to evaluate possible associated factors. Data collection included the application of a questionnaire and the collection of cervical swabs from pregnant women. Species identification was performed by real-time PCR. The overall prevalence of *Mollicutes* was 60.97%. 55.9% of pregnant women were colonized by *Ureaplasma* spp., and 19.51% by *Mycoplasma* spp. The prevalence rates by species were 48.78% for *U. parvum*, 11.59% for *U. urealyticum*, 18.9% for *M. hominis*, and 1.22% for *M. genitalium.* Age, 12 years of schooling or more, age at first sexual intercourse up to 14 years, third trimester of pregnancy, having undergone infertility treatment, presence of STI, and groin lymph nodes were associated with a higher prevalence of microorganisms. The results presented are of utmost importance for understanding the prevalence of these microorganisms, the characteristics of colonized pregnant women, and planning screening strategies and interventions that minimize the negative impacts of these infections.

## Introduction

Infections by *Ureaplasma urealyticum*, *Ureaplasma parvum*, *Mycoplasma genitalium*, and *Mycoplasma hominis*, species of the *Mollicutes* class and collectively referred to as genital mycoplasmas, have gained increasing attention due to their impacts on women’s health. These microorganisms commonly colonize the urogenital tract, often without symptoms, but are associated with several clinical conditions such as urethritis, cervicitis, and pelvic inflammatory disease [1]. During pregnancy, colonization by mycoplasmas may be related to adverse outcomes, including spontaneous abortion, preterm birth, premature rupture of membranes, chorioamnionitis, and low birth weight [2]. Furthermore, vertical transmission of mycoplasmas during childbirth can cause neonatal infections, increasing neonatal morbidity and mortality [3].

The appropriate diagnosis and treatment of genital mycoplasma infections are challenging due to the insidious nature of these bacteria and their resistance to common antibiotics [4]. The most sensitive detection of these pathogens involves molecular biology techniques, which are not always available in resource-limited healthcare settings. In addition, the individual pathogenic potential of genital mycoplasma species, their synergy with other microorganisms, their interaction with the host immune system, and the effects that microbial load has on the change from mere colonization to infection with clinical repercussions are still unclear [5].

This study aims to contribute to the epidemiological knowledge of these infections, offering data that can guide prevention, diagnosis, and treatment. Although studies are showing the prevalence and adverse effects of genital mycoplasma infection in pregnant women, the data are still insufficient to clarify all aspects of this interaction, and it is crucial to investigate the prevalence of *Mollicutes* in pregnant women, evaluate the associated risk factors and the clinical implications of these infections [6]. Thus, the objective of the study was to investigate the presence of four species of *Mollicutes* (*M. hominis*, *M. genitalium*, *U. parvum*, and *U. urealyticum*) in the cervical region of pregnant women attended for high-risk prenatal care at a maternal and child referral unit in southwestern Bahia, Brazil, and to evaluate possible associated factors.

## Methods

### Study design

This cross-sectional study aimed to determine the prevalence of genital mycoplasma colonization in pregnant women. Researchers from the Federal University of Bahia and the State University of Santa Cruz collected data and biological material from pregnant women attending high-risk prenatal care at a maternal and child health unit in Vitória da Conquista, Bahia, Brazil.

### Population

Pregnant women aged 18 years or older, at any gestational age, and meeting the criteria for high-risk prenatal care by the Technical Manual for High-Risk Pregnancy of the Brazilian Ministry of Health (Brazil, 2022), such as obesity, repeated spontaneous abortion, preterm birth in a previous pregnancy, hypertension or diabetes before pregnancy, infectious diseases during pregnancy and uterine malformations, took part in the study. Pregnant women who used antibiotics to treat infection in the last 14 days before collection were excluded. The sample consisted of 164 pregnant women treated between November 2021 and November 2022.

### Ethical procedures

This research complied with the provisions of Resolutions 466/12 and 510/2016 of the National Health Council, which regulate research involving human beings. It was carried out only after approval by the Ethics and Research Committee of the Multidisciplinary Institute of Health of the Federal University of Bahia – IMS/UFBA, under CAAE: 40117020.4.0000.5556. The researchers approached the participants empathetically, and the objectives of the study and the details regarding data collection and biological materials that would be collected were presented through the reading of the Free and Informed Consent Form (FICF); after consent, this was then signed by the participant, who kept a copy.

### Data and sample collection

Data collection consisted of applying a semi-structured questionnaire to identify the patient’s epidemiological profile, which was divided into modules and adapted from the study by Campos et al. [7]. In addition, a cervical sample was collected by the gynecologists/obstetricians participating in the research and included in the routine of the health service. The order of collection of the cervical sample and questionnaire was conducted not to hinder the flow of clinical care in the health service. The cervical material was collected with a sterile cotton swab in the doctor’s office, with the aid of a speculum, packaged in a Stuart transport medium, and transported in isothermal boxes to the Microbiology and Immunology laboratory of the Multidisciplinary Institute of Health, Federal University of Bahia. Upon arrival at the laboratory, the swabs were packaged in 15 ml Falcon tubes, incorrectly identified as containing 5 ml of PBS. The samples were then homogenized, aliquoted into 1.5 ml microtubes, and stored at −20 °C until processing.

### DNA extraction and identification by qPCR

For microbial identification of cervical samples, genomic DNA was initially extracted using the standardized boiling and PBS method [8]. DNA aliquots were then subjected to quantification and quality analysis by spectrophotometry in a NanoDrop (Thermo ScientificTM 5 NanoDrop 2000) at OD 260/280, also observing whether there was the presence of contaminants, such as lipids and proteins. Then, a real-time polymerase chain reaction was performed in StepOne Plus (Life Technologies) with a final volume of 25 μl and Master Mix (Thermo Fisher Scientific, Waltham, MA, United States). To identify the microbial species, TaqMan probes were used, following a basic amplification protocol for the species *U. urealyticum* [9], *U. parvum* [9], *M. hominis* [10], and *M. genitalium* [11]. Positive control (DNA extracted from the culture of microorganisms isolated and characterized in previous studies by the same research group and kept at −70 °C in PBS medium), negative control (no DNA), and samples were included.

### Statistical analysis

Initially, descriptive analysis of all variables was performed using absolute and relative frequencies (%). The prevalence of all outcomes was estimated, along with their 95% confidence intervals (95% CI). A bivariate analysis of the explanatory variables and outcomes was performed. Differences between categorical variables and the occurrence of each outcome were tested using Pearson’s chi-squared test or Fisher’s exact test. In all tests, *p* < 0.05 was considered significant. The prevalence ratio (PR) and its respective 95% confidence interval (95% CI) were estimated using Poisson regression with robust variance.

Multivariate analysis was performed for the following outcomes: presence of *Mollicutes*, presence of *Ureaplasma* spp., presence of *Mycoplasma* spp., presence of *U. urealyticum*, presence of *U. parvum*, and presence of *M. hominis.* For this stage, the variables that obtained a *p*-value <0.20 in the bivariate analysis were selected. The multivariate models were analyzed using the backward stepwise method for variable selection and compared using the Akaike information criterion (AIC). The adequacy of the final model was assessed using the chi-squared test (goodness-of-fit). Only variables with a *p*-value <0.05 remained in the final statistical model. The statistical software STATA version 16.1 (Stata Corporation, College Station, USA) was used for all analyses.

## Results

There was variation in the sample size, as collecting information from the questionnaire for five pregnant women was impossible. For this reason, the data presented in Table 1 refer to the sample number of 159 pregnant women who collected swabs and questionnaires. Most study participants were between 20 and 34 years old, had a partner at the time of the study, were from urban areas, reported being non-black, had 12 or more years of education, and had an income of less than one minimum wage. Most were in the 3rd trimester of pregnancy, did not practice physical activity (aerobic exercise or weight training at least three times a week), and did not drink alcohol. Regarding gynecological and obstetric history, most reported not having had fibroids, polycystic ovaries, or other gynecological alterations, had not had an ectopic pregnancy, and had not undergone infertility treatment. Regarding STI history (HIV, syphilis, hepatitis B and C, HPV, genital herpes, gonorrhea, chlamydia and trichomoniasis), most reported not having had an STI in the past, had not undergone treatment for STI, and current STI tests (performed at the health service and which did not include mycoplasmas for either the pregnant woman or the partner) were negative. Most had their first sexual intercourse after the age of 15, had more than one sexual partner during their lifetime, reported pain during sexual intercourse, reported bleeding during sexual intercourse, and did not use condoms or used them inconsistently. More than half had undergone a gynecological examination within 1 year before the survey and used contraceptives. Regarding obstetric history, the number of pregnancies was relatively homogeneous: 1, 2, or 3 or more. Most had no miscarriages, preterm births, or stillbirths. Regarding the number of expected prenatal consultations, most had six or more consultations and had not used antibiotics in the last 3 months. Most pregnant women had one or two or more symptoms at some point during pregnancy. More than half of the pregnant women had vaginal discharge as a symptom. Regarding information about their partners, most had not undergone treatment for infertility or STIs.

**Table 1.** Characteristics of pregnant women receiving high-risk prenatal care in southwestern Bahia (*n* = 159). Brazil, 2021–2022

Variables	*n* ^a^	%	CI 95%^b^
Age			
Up to 19 years	22	13.8	09.2–20.2
20 to 34 years	91	57.2	49.4–64.7
35 years and over	46	28.9	22.4–36.5
Marital status			
With partner	145	91.2	85.6–94.7
Without partner	14	8.8	5.3–14.4
Origin			
Urban area	119	74.8	67.5–81.0
Rural area	40	25.2	19.0–32.5
Color			
Black	35	22.0	16.2 -29.2
Non-black	124	78.0	70.8–83.8
Education			
Up to 11 years	65	40.9	33.4–48.7
12 years or older	94	59.1	51.3–66.5
Income			
<1 minimum wage	85	53.8	46.0–61.5
1 minimum wage or more	73	46.2	38.5–54.1
Gestational age			
1st trimester	14	09.0	05.4–14.7
2nd trimester	66	42.6	35.0–50.5
3nd trimester	75	48.4	40.5–56.3
Physical activity			
No	125	78.6	71.5–84.3
Yes	34	21.4	15.6–28.5
Use of alcoholic beverages			
No	150	94.3	89.4–97.0
Yes	9	5.7	2.9–10.7
Myoma			
No	143	89.9	84.1––93.8
Yes	16	10.1	6.2–15.8
Polycystic ovaries			
No	136	85.5	79.1––90.2
Yes	23	14.5	9.8–20.9
Other gynecological alteration			
No	146	91.8	86.4–95.2
Yes	13	8.2	4.8–13.6
Had any STIs^c^			
No	144	90.6	84.9–94.2
Yes	15	9.4	5.7–15.1
Underwent treatment for STI			
No	145	91.2	85.6–94.7
Yes	14	8.8	5.3–14.4
Ectopic pregnancy			
No	153	96.2	91.8–98.3
Yes	6	3.8	1.7–8.2
Infertility treatment			
No	149	93.7	88.6–96.6
Yes	10	6.3	3.4–11.3
STI testing during prenatal care			
Negative	153	96.2	91.8–98.3
Positive	6	3.8	1.7–8.2
Age of first sexual intercourse			
Up to 14 years	26	17.3	12.0–24.3
15 years or older	124	82.7	75.7–88.0
Had more than 1 sexual partner			
No	46	29.9	23.1–37.6
Yes	108	70.1	62.4–76.9
Pain during sexual intercourse			
No	28	18.1	12.7–25.0
Yes	127	81.9	75.0–87.3
Bleeding during intercourse			
No	148	95.5	90.8–97.9
Yes	7	4.5	2.1–9.2
Condom use			
No/Sometimes	121	78.1	70.8–84.0
Yes	34	21.9	16.0–29.2
Year of last gynecological examination			
In the last year	87	54.7	46.9–62.3
More than 1 year	72	45.3	37.7–53.1
Use of contraceptives			
No	77	49.0	41.2–56.9
Yes	80	51.0	43.1–58.7
Number of pregnancies			
1	51	32.1	25.2–39.8
2	45	28.3	21.8–31.8
3 or more	63	39.6	32.3–47.5
Abortion			
None	112	70.9	63.3–77.5
1 or more	46	29.1	22.5–36.7
Preterm birth			
None	128	81.0	74.0–86.4
1 or more	30	19.0	13.6–25.9
Stillborn			
None	131	83.4	76.7–88.5
1 or more	26	16.6	11.5–23.3
Expected prenatal appointments			
≥6 consultations	117	76.5	69.0–82.5
<6 consultations	36	23.5	17.4–30.9
Use of antibiotics in the last 3 months			
No	102	64.2	56.3–71.3
Yes	57	35.9	28.7–43.6
Presence of symptoms during pregnancy			
None	29	18.1	12.9–24.9
1	55	34.4	27.4–42.1
2 or more	76	47.5	39.8–55.3
Vaginal discharge			
No	76	47.8	40.0–55.6
Yes	83	53.2	44.4–60.0
Itching			
No	136	85.5	79.1–90.2
Yes	23	14.5	9.8–20.9
Vaginal erythema			
No	154	96.9	92.6–98.7
Yes	5	3.1	1.3–7.4
Pelvic pain			
No	84	52.8	45.0–60.0
Yes	75	47.2	39.5–55.0
Genital warts			
No	158	99.4	95.6–99.9
Yes	1	0.6	0.0–4.3
Genital vesicles			
No	156	98.1	94.3–99.4
Yes	3	1.9	0.6–5.7
Genital wounds			
No	157	99.4	95.6–99.9
Yes	1	0.6	0.0–4.4
Pain when urinating			
No	133	83.7	77.0–88.6
Yes	26	16.4	11.3–23.0
Swollen glands in the groin			
No	151	94.4	89.5–97.0
Yes	9	5.6	2.93–10.5
Other gynecological alteration			
No	125	78.1	71.0–83.0
Yes	35	21.9	16.10–29.0
Partner treatment for infertility			
No	149	93.7	88.6–96.6
Yes	10	6.3	3.4–11.3
Partner treatment for STI			
No	149	94.3	89.4–97.0
Yes	9	5.7	3.0–10.6

a *n* = absolute frequency.

b IC95%: confidence interval 95%.

c STI = Sexually transmitted infection.

**Figure 1.**
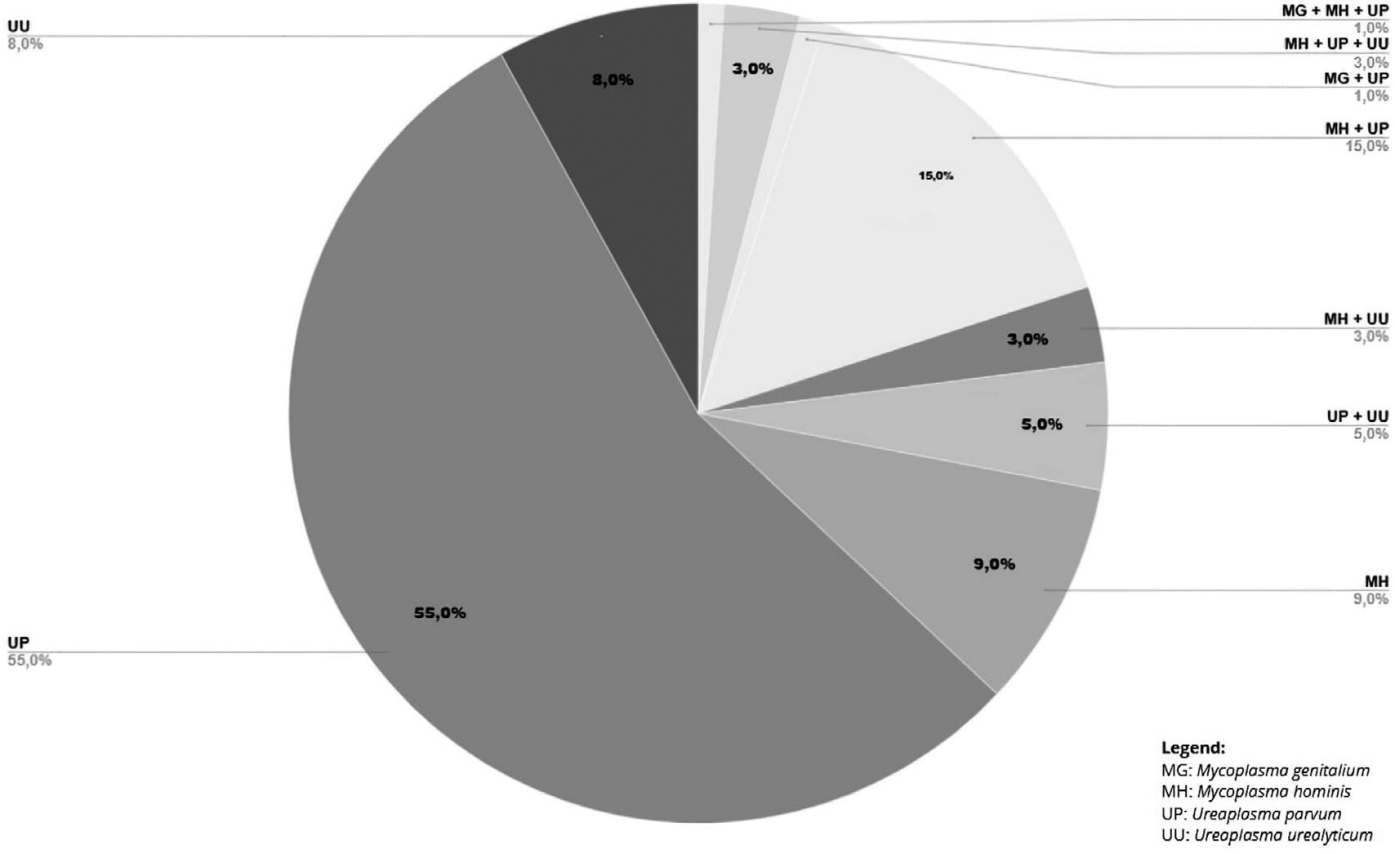
Coinfection between Mollicutes species in pregnant women receiving high-risk prenatal care in southwesternBahia (n = 100). Brazil, 2021-2022.

The overall prevalence of *Mollicutes* was 60.97% (*n* = 100). 55.9% (*n* = 91) of the pregnant women were colonized by *Ureaplasma* spp., and 19.51% (*n* = 32) were colonized by *Mycoplasma* spp. The prevalences by species were: 48.78% (*n* = 80) *U. parvum*, 11.59% (*n* = 19) *U. urealyticum*, 18.9% (*n* = 31) *M. hominis*, and 1.22% (*n* = 2) *M. genitalium* (Table 2). Coinfection between *Mollicutes* species occurred in 17.07% (*n* = 28), and isolated infection occurred in 43.9% (*n* = 72) of the participants (Table 2). Figure 1 shows the detection percentage for isolated infection and coinfection among positive cases.

**Table 2.** Prevalence of *Mollicutes* colonization in pregnant women receiving high-risk prenatal care in southwestern Bahia (*n* = 164). Brazil, 2021–2022

Variables	*n* ^a^	%	CI 95%^b^
*Mycoplasma* spp.	32	19.5	14.1–26.3
*Mycoplasma genitalium*	2	1.2	0.3–4.8
*Mycoplasma hominis*	31	18.9	13.6–25.7
*Ureaplasma* spp.	91	55.9	47.7–63.0
*Ureaplasma urealyticum*	19	11.6	7.5–17.5
*Ureaplasma parvum*	80	48.8	41.2–56.4
*Mollicutes* infection			
None	64	39.2	31.8–46.7
Isolated	72	43.9	36.4–51.6
Co-infection	28	17.1	12.0–23.7

a *n* = absolute frequency.

b CI95%: confidence interval 95%.

Table 3 presents the results that showed statistical significance in the bivariate analysis for the prevalence of *Mollicutes*, *Mycoplasma* spp., and *Ureaplasma* spp. Table 4 shows the statistical significance of the bivariate analysis for *Ureaplasma* spp., *U. parvum*, and *U. urealyticum.* Table 5 presents the statistical significance data from the bivariate analysis for *Mycoplasma* spp., *M. genitalium*, and *M. hominis.* The complete data from the bivariate analysis can be found in Supplementary Tables S1, S2, and S3, which are included in the Supplementary Materials.

**Table 3.** Bivariate analysis for the prevalence of *Mollicutes*, *Mycoplasma* spp. and *Ureaplasma* spp. in pregnant women receiving high-risk prenatal care in southwestern Bahia (*n* = 164). Brazil, 2021–2022

Variables	*Mollicutes*				*Mycoplasma* spp.					*Ureaplasma* spp.					
	*n* ^a^	P(%)^b^	*p*-value	PR^c^	CI 95%^d^	*n* ^a^	P(%)^b^	*p*-value	PR^c^	CI 95%	*n* ^a^	P(%)^b^	*p*-value	PR^c^	CI 95%
Age			**0.031**					**0.001**					0.237		
Up to 19 years old	19	86.40		1.00	–	11	50.00		1.00	–	16	72.73		1.00	–
20–34	51	56.00		0.65	0.51–0.83	13	14.29		2.88	1.35–6.14	48	52.75		0.73	0.90–1.85
35 or more	29	63.00		0.73	0.55–0.96	8	17.39		0.82	0.37–1.84	26	56.52		0.78	0.68–1.29
Marital status			0.459					**0.037** ^e^					0.241		
With partner	89	61.38		1.00	–	32	22.07		1.00	–	80	55.17		1.00	–
Without partner	10	71.43		1.16	0.81–1.66	**	**	**	**	**	10	71.43		1.29	0.90–1.86
Gestational age			0.749					**0.024**					0.356		
1st trimester	10	71.43		1.00	–	**	**		**	**	10	71.43		1.00	–
2 nd trimester	40	60.61		0.85	0.57–1.24	10	15.15		**	**	39	59.09		0.83	0.56–1.22
3rd trimester	47	62.67		0.88	0.60–1.28	21	28.00		**	**	39	52.00		0.73	0.49–1.08
Myoma			**0.031**					0.335^e^					0.104		
No	93	65.03		1.00	–	30	20.98		1.00	–	84	58.74		1.00	–
Yes	6	37.50		0.58	0.30–1.10	2	12.50		0.60	0.16–2.27	6	37.5		0.64	0.33–1.22
Polycystic ovaries			**0.013**					0.488^e^					**0.022**		
No	90	66.18		1.00	–	28	20.59		1.00	–	82	60.29		1.00	–
Yes	9	39.13		0.59	0.35–1.00	4	17.39		0.84	0.33–2.19	8	34.78		0.58	0.32–1.03
Age of first sexual intercourse			0.176					**0.030**					0.117		
≥15 years	73	58.87		1.00	–	20	16.13		1.00	–	65	52.42		1.00	–
Up to 14 years	19	73.08		1.24	0.94–1.63	9	34.62		2.15	1.10–4.18	18	69.23		1.32	0.97–1.80
Vaginal discharge			0.665					0.780					0.995		
Partner treatment for infertility			0.034					0.495^e^					**0.000**		
No	95	63.76		1.00	–	29	20.42		1.00	–	87	61.27		1.00	–
Yes	3	30.00		0.47	0.18–1.23	3	16.67		0.82	0.28–2.42	3	16.67		0.27	0.10–0.77

a *n* = Absolute frequency.

b P = Prevalence.

c PR = Crude prevalence ratio.

d CI95%: Confidence interval 95%.

e Fisher’s exact test.

** Statistical analysis was not possible.

Statistically significant values are highlighted in bold.

**Table 4.** Bivariate analysis for the prevalence of *Ureaplasma* spp., *Ureaplasma parvum* and *Ureaplasma urealyticum* in pregnant women receiving high-risk prenatal care in southwestern Bahia (*n* = 164). Brazil, 2021–2022

Variables	*Ureaplasma* spp.				*U. urealyticum*					*U. parvum*					
	*n* ^a^	P(%)^b^	*p*-value	PR^c^	CI 95%^d^	*n* ^a^	P(%)^b^	*p*-value	PR^c^	CI 95%^d^	*n* ^a^	P(%)^b^	*p*-value	PR^c^	CI 95%^d^
Polycystic ovaries			**0.022**					0.498^e^					0.039		
No	82	60.29		1.00		16	11.76		1.00		73	53.68		1.00	
Yes	8	34.78		0.58	0.32–1.03	2	8.70		0.74	0.18–3.02	7	30.43		0.58	0.30–1.07
STI Treatment			0.241					**0.033**					0.274		
No	10	71.43		1.00	–	4	28.57		1.00	–	9	64.29		1.00	–
Yes	80	55.17		0.77	0.54–1.11	14	9.66		0.34	0.13–0.89	71	48.97		0.76	0.50–1.17
Ectopic pregnancy			0.527^e^					**0.020** ^e^					**0.014** ^e^		
No	87	56.86		1.00	–	15	9.80		1.00	–	80	52.29		1.00	–
Yes	3	50.00		0.88	0.39–1.99	3	50.00		5.1	2.00–13.00	**	**		**	**
Use of contraceptives			0.119					0.678					**0.031**		
No	48	62.34		1.00	–	8	10.39		1.00	–	45	58.44		1.00	–
Yes	40	50.00		0.80	0.61–1.06	10	12.50		1.20	0.50–2.90	33	41.25		0.71	0.51–0.98
Vaginal discharge			0.995					**0.028**					0.940		
No	43	56.58		1.00	–	13	17.11		1.00	–	38	50.00		1.00	–
Yes	47	56.63		1.00	0.76–1.32	5	6.02		0.35	0.13–0.94	42	50.60		1.01	0.74–1.38
Partner treatment for infertility			**0.000**					0.102^e^					**0.003**		
No	87	61.27		1.00	–	18	12.68		1.00	–	77	54.23		1.00	-
Yes	3	16.67		0.27	0.10–0.77	**	**		**	**	3	16.67		0.31	0.11–0.88

a *n* = Absolute frequency.

b P = Prevalence.

c PR = Crude prevalence ratio.

d CI95%: Confidence interval 95%.

e Fisher’s exact test; **Statistical analysis was not possible.

Statistically significant values are highlighted in bold.

**Table 5.** Bivariate analysis for the prevalence of *Mycoplasma* spp., *Mycoplasma genitalium* and *Mycoplasma hominis* in pregnant women receiving high-risk prenatal care in southwestern Bahia (*n* = 164). Brazil, 2021–2022

Variables	*Mycoplasma* spp.				*M. genitalium*						*M. hominis*				
	*n* ^a^	P(%)^b^	*p*-value	PR^c^	CI 95%^d^	*n* ^a^	P(%)^b^	*p*-value	PR^c^	CI 95%^d^	*n* ^a^	P(%)^b^	*p*-value	PR^c^	CI 95%^d^
Age			**0.001**					0.341^e^					**0.000**		
Up to 19 years old	11	50.00		1.00	–	1	4.55		1.00	–	11	50.00		1.00	–
20–34	13	14.29		2.88	1.35–6.14	1	1.10		**	**	12	13.19		2.88	1.35–6.14
35 or more	8	17.39		0.82	0.37–1.84	**	**		**	**	8	17.39		0.76	0.33–1.73
Gestational age			**0.024**					0.585^e^					**0.036**		
1st trimester	**	**		**	**	**	**		**	**	**	**		**	**
2 nd trimester	10	15.15		**	**	**	**		**	**	10	15.15		**	**
3rd trimester	21	28.00		**	**	2	2.67		**	**	20	26.67		**	**
Physical activity			**0.298**					0.617^e^					0.247		
No	23	18.40		1.00	–	2	1.60		1.00	–	22	17.60		1.00	–
Yes	9	26.47		1.44	0.73–2.82	**	**		**	**	9	26.47		1.50	0.76–2.96
Age of first sexual intercourse			**0.030**					0.682^e^					**0.026** ^e^		
≥15 years	20	16.13		1.00	–	2	1.61		1.00	–	19	15.32		1.00	–
Up to 14 years	9	34.62		2.15	1.10–4.18	**	**		**	**	9	34.62		2.26	1.15–4.43

a *n* = Absolute frequency

b P = Prevalence.

c PR = Crude prevalence ratio.

d CI95%: Confidence interval 95%.

e Fisher’s exact test.

** Statistical analysis was not possible.

Statistically significant values are highlighted in bold.

After multivariate analysis, the following factors were associated with a higher prevalence of *Mollicutes*: 12 or more years of schooling (PR = 1.36, CI95% = 1.04–1.78), age at first sexual intercourse up to 14 years (PR = 1.41, CI95% = 1.07–1.84), and infertility treatment (PR = 0.32, CI95% = 0.14–0.75). For *Ureaplasma* spp.: age at first sexual intercourse up to 14 years (PR = 1.37, CI95% = 1.03–1.81) and infertility treatment (PR = 0.27, CI95% = 0.09–0.76). For *Mycoplasma* spp., being between 20 and 34 years of age (PR = 2.61, CI95% = 1.10–6.20). For *U. urealyticum*: being in the 3rd trimester of pregnancy (PR = 0.24, CI95% = 0.08–0.77), having had STIs (PR = 2.58, CI95% = 1.09–6.12), and having groin lymph nodes (PR = 3.07, CI95% = 1.04–9.04). For *U. parvum*: 12 years of schooling or more (PR = 1.60, CI95% = 1.11–2.30), age at first sexual intercourse up to 14 years (PR = 1.60, CI95% = 1.13–2.26), and infertility treatment (PR = 0.30, CI95% = 0.11–0.82). For *M. hominis*: age between 20 and 34 years (PR = 2.57, CI95% = 1.08–6.11). These data are presented in Table 6.

**Table 6.** Multivariate analysis for the prevalence of *Mollicutes*, *Mycoplasma* spp., *Ureaplasma* spp., *Ureaplasma urealyticum*, *Ureaplasma parvum* and *Mycoplasma hominis* in pregnant women receiving high-risk prenatal care in southwestern Bahia (*n* = 164). Brazil, 2021–2022

Mollicutes			
Variables	PR^a^	CI 95%^b^	*p*-value
Education			0.026
12 years or older	1.36	1.04–1.78	
Up to 11 years	1.00	–	
Age of first intercourse			0.013
Up to 14 years	1.41	1.07–1.84	
≥15 years	1.00	–	
Infertility treatment			0.009
Yes	0.32	0.14–0.75	
No	1.00	–	

*Ureaplasma* ssp.			
Variables	PR^a^	CI 95%^b^	*p*-value
Age of first intercourse			0.032
Up to 14 years	1.37	1.03–1.81	
≥15 years	1.00	–	
Infertility treatment			0.013
Yes	0.27	0.09–0.76	
No	1.00	–	

*Mycoplasma* ssp.			
Variables	PR^a^	CI 95%^b^	*p*-value
Age			0.029
20–34 years old	2.61	1.10–6.20	
Up to 19 years old	1.00	–	
Age of first intercourse			0.228
Up to 14 years	1.51	0.77–2.98	
≥15 years	1.00	–	

*U. urealyticum*			
Variables	PR^a^	CI 95%^b^	*p*-value
Gestational age			0.016
3rd trimester	0.24	0.08–0.77	
1st trimester	1.00	–	
Had STIs			0.031
Yes	2.58	1.09–6.12	
No	1.00	–	
Groin lymph nodes			0.042
Yes	3.07	1.04–9.04	
No	1.00	–	

*U. parvum*			
Variables	PR^a^	CI 95%^b^	*p*-value
Education			0.011
12 years or older	1.60	1.11–2.30	
Up to 11 years	1.00	–	
Age of first intercourse			0.008
Up to 14 years	1.60	1.13–2.26	
≥15 years	1.00	–	
Infertility treatment			0.018
Yes	0.30	0.11–0.82	
No	1.00	–	

*M. hominis*			
Variables	PR^a^	CI 95%^b^	*p*-value
Age			0.033
20–34 years old	2.57	1.08–6.11	
Up to 19 years old	1.00	–	
Age of first intercourse			0.193
Up to 14 years	1.58	0.80–3.14	
≥15 years	1.00	–	

a PR = Adjusted prevalence ratio.

b CI95%: Confidence interval 95%.

## Discussion

The prevalence of genital mycoplasmas in pregnant women has been reported in studies conducted in other countries and, to a lesser extent, in Brazil [12–19]. Some of these studies have attempted to associate risk factors for infection with adverse outcomes during pregnancy. However, it is still unclear whether all species are directly involved in the infection, whether the bacterial load is decisive, whether the crucial point is the interaction with other microorganisms, or whether it depends on the host response [6]. In Brazil, *Mollicutes* infections are not part of the standard STI screening protocol for pregnant women, and, in the absence of rapid diagnostic tests and difficulty in accessing molecular tests, syndromic treatment has been the practice. This was the first study to assess the prevalence of *Mollicutes* in pregnant women receiving high-risk prenatal care in the interior of Bahia.

The overall prevalence of *Mollicutes* found in our study (60.97%) was higher than the 9.3% reported in the survey by Peretz et al. [16] carried out with 214 pregnant women in Israel, in which vaginal swab samples and detection by culture and PCR were used. The use of vaginal swab in the study above may explain the differences mentioned, since a higher rate of colonization by *Mollicutes* in the cervical region is expected, as occurred in our study. The prevalence was also higher than that observed in the study by Lee et al. [13], who detected 571 cases of MH and UU among 1035 participants in South Korea. However, this study used culture as the detection method, and molecular detection, compared to culture, is expected to provide a higher detection rate due to the higher sensitivity of the technique. By species, we detected a higher prevalence of *U. parvum*, which is in agreement with the study by Payne et al. [15] carried out in Australia, which found a higher prevalence of *U. parvum* (between 35% and 63%) at three different times during pregnancy. This higher prevalence of *U. parvum* was not observed in studies such as that of Abdelaziz et al. [12], which showed a higher prevalence of *M. hominis*, and that of Lee et al. [13], which found a higher prevalence of *U. urealyticum.*

The present study also identified a high co-infection by at least two species, emphasizing co-infection between *M. hominis* and *U. urealyticum.* A retrospective cohort study carried out by Jeon et al. [20] in South Korea included 1381 pregnant women admitted to a high-risk unit and demonstrated that the group with genital colonization by mycoplasmas had a higher risk of premature rupture of membranes and chorioamnionitis compared to the group without colonization (42.4% vs. 35.6%, *p* = 0.013). In an extensive multicenter study with 3643 participants in Austria, Rittenschober-Böhm et al. [21] investigated *U. parvum* serovars in the vaginal region and their relationship with spontaneous preterm birth. In this study, in the samples positive for UP (37%, *n* = 1347), serovar 3 was the most common isolate, and there was a significantly increased risk of preterm birth at low (<32 weeks) and extremely low (<28 weeks) gestational age. The high prevalence of genital mycoplasmas in pregnant women deserves attention since several studies have shown an association of these microorganisms with adverse pregnancy outcomes.

Still, about premature birth, the study by Vouga et al. [22] in Switzerland evaluated colonization by mycoplasmas and subsequent treatment in 5.377 pregnant women. 2.259 women (42%) had a positive culture for *Ureaplasma* spp. or *M. hominis*, representing significant colonization (percentage of positive cultures), even using a less sensitive method. Women colonized by genital mycoplasmas and treated demonstrated lower premature birth rates (*Ureaplasma* spp., *p* 0.024; *M. hominis*, *p* 0.001). Some studies have shown that genital mycoplasma infection may play an essential role in the etiology of spontaneous abortion [18, 19, 23–25, 26], premature rupture of membranes [27–29], chorioamnionitis [30–32], intra-amniotic infection [33–35] and other obstetric complications [36–38].

The prevalence of *Mollicutes*, *Ureaplasma* spp., and *U. parvum* in the present study was associated with pregnant women who had their first sexual intercourse at up to 14 years of age. Although we did not find studies in the literature that associated this same risk factor for infection with the *Mollicutes* class in pregnant women, the study by Lee et al. [13] found a high prevalence (88.2%) of *Mollicutes* in pregnant women aged between 15 and 19 years, which may be a reflection of the early onset of sexual activity, drawing attention to the need for more excellent care with pregnant adolescents. Studies indicate a strong association between the early onset of sexual activity and STIs [39], which implies increased costs for the management of these infections in health services.

Our study observed an association between infertility treatment and the prevalence of *Mollicutes*, *Ureaplasma* spp., and *U. parvum.* Regarding infertility, many studies show that genital mycoplasmas influence male and female fertility. The inflammatory processes triggered by these pathogens can lead to pelvic inflammatory disease, deterioration of spermatogenesis, obstruction of the seminal tract, agglutination of motile sperm, induction of apoptosis, production of immobilization factors, and impairment of the acrosome region [40–42] and some mycoplasma strains resistant to macrolide treatment have already been reported [43]. By correlating this information with the results obtained in our study, it is possible to assume that having undergone treatment implies that, at some point before pregnancy, there was an investigation for infertility, with one of the potential causes being Mycoplasma infection. Once the treatment was performed, the infection was resolved. The woman was able to become pregnant. However, mycoplasma infection is only one of the possible causes of infertility, and we did not have information on the reason for the women who underwent treatment.

There are also conflicting studies in the literature, such as that by Günyeli et al. [44], which indicate no differences between fertile and infertile couples regarding Mycoplasma infection. Even so, the association between infertility treatment and *Mollicutes* infection should be monitored in more depth since professionals working in reproductive medicine are aware of the high rate of couples with difficulty getting pregnant due to infection by some microorganisms, including mycoplasmas. In cases such as couples with infertility or pregnant women with complications in previous pregnancies in which *Mollicutes* were isolated, screening and treatment protocols for these infections could be implemented, reducing the negative impacts on health and costs. Although some studies have not found evidence to screen and treat colonized patients without complications, there is still a need for more studies with specific patient groups, such as pregnant women.

Regarding the association between *Mollicutes* and women having previously had STIs, there are studies in the literature that reveal that mycoplasmas are more frequent in HIV-infected patients, as shown in the systematic review by Boujemaa, Singh-Suri, and Kaur [45]. In our study, HIV infection was not reported in any of the participants. The study by Koch et al. [46] showed that in both men and women, infection by *Trichomonas vaginalis* increases colonization by *M. hominis* and *U. urelyticum.* A high prevalence of co-infection by HPV and *U. parvum* [47] and *U. urealyticum* [48] was also observed. Infection by *M. genitalium* is considered an emerging STI with controversial management [49]. In recent decades, there has been a growing association between Mycoplasma infections and other STIs; however, epidemiological surveillance data provided by the Brazilian Ministry of Health [50] are limited to HIV/AIDS, hepatitis, and syphilis. Estimating the prevalence of these infections worldwide is necessary to clarify diagnostic gaps, design control programs, and allocate health resources.

In pregnant women, data on STIs and mycoplasmas are even more limited, which is a concern since these infections, when undetected and untreated during pregnancy, are associated with adverse maternal and neonatal outcomes. The pregnancy condition is known to be immunotolerant, which may contribute to susceptibility to pathogens. In addition, pregnant women report lower rates of condom use, although less sexual activity and less risky sexual behaviors may offset this risk [51]. Although there are reports of *M. hominis* and herpes simplex infection in pregnant women [52] and some studies, such as that by Stafford et al. [53], show the association of *M. genitalium* with *Chlamydia trachomatis*, the existing data are not yet sufficient to demonstrate the cause-and-effect relationship in these associations, especially regarding other species of genital mycoplasmas. In our study, we observed that pregnant women who had some STIs had a higher risk of infection by *U. urealyticum.* This finding opens the possibility for further research to try to determine which specific STIs are associated with infection by *U. urealyticum* and other genital mycoplasmas.

Our study found a positive association between having 12 or more years of education and infection by *Mollicutes* and *U. parvum.* This finding differs from that found in the study by Jeon et al. [20], which showed that a lower level of education tends to be associated with genital colonization by mycoplasmas in high-risk pregnant women. For the gestational age variable, we observed in our research that pregnant women in the 3rd trimester of pregnancy had a lower chance of infection by *U. urealyticum*, which contrasts with the finding of the work by Abdelaziz et al. [12] in which of the 200 pregnant women, 176 (88%) presented positivity for some bacteria in the vaginal region, most of them in the 3rd trimester of pregnancy (71.6%) and also contrasts with the study by Payne et al. [15] which demonstrated that the prevalence of UU and UP were similar in the three moments of pregnancy (1st, 2nd and 3rd trimesters), allowing us to understand that the colonization status is preserved during pregnancy. In the present study, we also observed that the presence of groin lymph nodes is positively associated with infection by *U. urealyticum.* However, we did not find other studies demonstrating this association for comparison purposes.

The results we present on the detection of *Mollicutes* in pregnant women have important implications for helping to understand the prevalence profile of these microorganisms in this population group. Since it has been demonstrated that mycoplasma infection has negative impacts on maternal and neonatal health, studies such as this provide data for the health system that help in the development of health policies aimed at screening and treating these infections, reducing health costs with the management of complications resulting from these infections. The high prevalence found in our study, associated with data from other studies, leads us to believe that if there were screening for genital mycoplasmas during prenatal care and subsequent treatment, adverse pregnancy outcomes would probably be reduced. Further research will be needed to confirm the prevalence and causal relationship and identify risk factors for *Mollicutes* infections in pregnant women. Studies must be well controlled and with different groups, which demonstrate the bacterial load, vaginal pH, quantity of *Lactobacillus*, elevation of cytokines, and clinical conditions of pregnant women. It is worth noting that many studies in the literature did not present consistent data and used methods that were not very sensitive for detecting microorganisms. Although we had limitations in our study, we associated important epidemiological variables and used the gold standard method for detecting mycoplasmas.

## Supporting information

10.1017/S0950268825100137.sm001 Santana et al. supplementary material Santana et al. supplementary material

## Data Availability

Raw data for this study are available upon request from the corresponding author.

## Supplementary material

The supplementary material for this article can be found at http://doi.org/10.1017/S0950268825100137.
