# Supplementary material for: Prevalence of Mollicutes in pregnant women undergoing high-risk prenatal care at a maternal and child reference unit in Bahia, Brazil
Source: Epidemiol Infect. 2025 Jun 25;153:e73. doi: 10.1017/S0950268825100137 (PMC12202237; doi:10.1017/S0950268825100137)
Supplement: Santana et al. supplementary material [file S0950268825100137sup001.docx]

**Table S1.** Bivariate analysis for the prevalence of *Mollicutes*, *Mycoplasma spp.* and *Ureaplasma spp.* in pregnant women receiving high-risk prenatal care in southwestern Bahia (n = 164). Brazil, 2021-2022.

|  | *Mollicutes* | | | | | *Mycoplasma spp*. | | | | | | *Ureaplasma spp*. | | | | | |
| --- | --- | --- | --- | --- | --- | --- | --- | --- | --- | --- | --- | --- | --- | --- | --- | --- | --- |
| Variables | n* | P(%)† | p-value | PR‡ | CI 95%§ | | n* | P(%)† | p-value | PR‡ | CI 95% | | n* | P(%)† | p-value | PR‡ | CI 95% |
| **Age** |  |  | **0,031** |  |  | |  |  | **0,001** |  |  | |  |  | 0,237 |  |  |
| Up to 19 years old | 19 | 86,40 |  | 1,00 | - | | 11 | 50,00 |  | 1,00 | - | | 16 | 72,73 |  | 1,00 | - |
| 20 - 34 | 51 | 56,00 |  | 0,65 | 0,51 - 0,83 | | 13 | 14,29 |  | 2,88 | 1,35 – 6,14 | | 48 | 52,75 |  | 0,73 | 0,90 - 1,85 |
| 35 or more | 29 | 63,00 |  | 0,73 | 0,55 - 0,96 | | 8 | 17,39 |  | 0,82 | 0,37 – 1,84 | | 26 | 56,52 |  | 0,78 | 0,68 - 1,29 |
| **Marital status** |  |  | 0,459 |  |  | |  |  | **0,037**^¶^ |  |  | |  |  | 0,241 |  |  |
| With partner | 89 | 61,38 |  | 1,00 | - | | 32 | 22,07 |  | 1,00 | - | | 80 | 55,17 |  | 1,00 | - |
| Without partner | 10 | 71,43 |  | 1,16 | 0,81 - 1,66 | | ** | ** | ** | ** | ** | | 10 | 71,43 |  | 1,29 | 0,90 - 1,86 |
| **Origin** |  |  | 0,472 |  |  | |  |  | 0,374 |  |  | |  |  | 0,330 |  |  |
| Rural area | 23 | 57,50 |  | 1,00 | - | | 10 | 25,00 |  | 1,00 | - | | 20 | 50,00 |  | 1,00 | - |
| Urban area | 76 | 63,87 |  | 1,11 | 0,82 - 1,50 | | 22 | 18,49 |  | 0,74 | 0,38 – 1,43 | | 70 | 58,82 |  | 1,18 | 0,83 - 1,66 |
| **Color** |  |  | 0,270 |  |  | |  |  | 0,329 |  |  | |  |  | 0,484 |  |  |
| Non-black | 80 | 64,52 |  | 1,00 | - | | 5 | 14,29 |  | 1,00 | - | | 18 | 51,43 |  | 1,00 | - |
| Black | 19 | 54,29 |  | 1,19 | 0,85 - 1,66 | | 27 | 21,77 |  | 1,52 | 0,63 – 3,68 | | 72 | 58,06 |  | 1,13 | 0,79 - 1,61 |
| **Education** |  |  | 0,137 |  |  | |  |  | 0,663 |  |  | |  |  | 0,217 |  |  |
| Up to 11 years | 36 | 55,38 |  | 1,00 | - | | 12 | 18,46 |  | 1,00 | - | | 33 | 50,77 |  | 1,00 | - |
| 12 or more | 63 | 67,02 |  | 1,21 | 0,93 - 1,57 | | 20 | 21,28 |  | 1,15 | 0,61 – 2,19 | | 57 | 60,64 |  | 1,19 | 0,89 - 1,60 |
| **Income** |  |  | 0,217 |  |  | |  |  | 0,629 |  |  | |  |  | 0,072 |  |  |
| Less than 1 minimum wage | 57 | 67,06 |  | 1,00 | - | | 16 | 18,82 |  | 1,00 | - | | 54 | 63,53 |  | 1,00 | - |
| 1 minimum wage or more | 42 | 57,53 |  | 0,86 | 0,67 - 1,09 | | 16 | 21,92 |  | 1,16 | 0,63 – 2,17 | | 36 | 49,32 |  | 0,78 | 0,58 - 1,03 |
| **Gestational age** |  |  | 0,749 |  |  | |  |  | **0,024** |  |  | |  |  | 0,356 |  |  |
| 1st trimester | 10 | 71,43 |  | 1,00 | - | | ** | ** |  | ** | ** | | 10 | 71,43 |  | 1,00 | - |
| 2nt trimester | 40 | 60,61 |  | 0,85 | 0,57 - 1,24 | | 10 | 15,15 |  | ** | ** | | 39 | 59,09 |  | 0,83 | 0,56 - 1,22 |
| 3rd trimester | 47 | 62,67 |  | 0,88 | 0,60 - 1,28 | | 21 | 28,00 |  | ** | ** | | 39 | 52,00 |  | 0,73 | 0,49 - 1,08 |
| **Physical activity** |  |  | 0,465 |  |  | |  |  | 0,298 |  |  | |  |  | 0,282 |  |  |
| No | 76 | 60,80 |  | 1,00 | - | | 23 | 18,40 |  | 1,00 | - | | 68 | 54,40 |  | 1,00 | - |
| Yes | 23 | 67,65 |  | 1,11 | 0,84 - 1,46 | | 9 | 26,47 |  | 1,44 | 0,73 – 2,82 | | 22 | 64,71 |  | 1,19 | 0,88 - 1,60 |
| **Alcoholic beverage** |  |  | 0,779 |  |  | |  |  | 0,574^¶^ |  |  | |  |  | 0,531 |  |  |
| No | 93 | 62,00 |  | 1,00 | - | | 30 | 20,00 |  | 1,00 | - | | 84 | 56,00 |  | 1,00 | - |
| Yes | 6 | 66,67 |  | 1,07 | 0,66 - 1,74 | | 2 | 22,22 |  | 1,11 | 0,31 – 3,95 | | 6 | 66,67 |  | 1,19 | 0,73 - 1,93 |
| **Myoma** |  |  | **0,031** |  |  | |  |  | 0,335^¶^ |  |  | |  |  | 0,104 |  |  |
| No | 93 | 65,03 |  | 1,00 | - | | 30 | 20,98 |  | 1,00 | - | | 84 | 58,74 |  | 1,00 | - |
| Yes | 6 | 37,50 |  | 0,58 | 0,30 - 1,10 | | 2 | 12,50 |  | 0,60 | 0,16 – 2,27 | | 6 | 37,5 |  | 0,64 | 0,33 - 1,22 |
| **Polycystic ovaries** |  |  | **0,013** |  |  | |  |  | 0,488^¶^ |  |  | |  |  | **0,022** |  |  |
| No | 90 | 66,18 |  | 1,00 | - | | 28 | 20,59 |  | 1,00 | - | | 82 | 60,29 |  | 1,00 | - |
| Yes | 9 | 39,13 |  | 0,59 | 0,35 - 1,00 | | 4 | 17,39 |  | 0,84 | 0,33 – 2,19 | | 8 | 34,78 |  | 0,58 | 0,32 - 1,03 |
| **Other gynecological changes** |  |  | 0,955 |  |  | |  |  | 0,492^¶^ |  |  | |  |  | 0,428 |  |  |
| No | 91 | 62,33 |  | 1,00 | - | | 30 | 20,55 |  | 1,00 | - | | 84 | 57,53 |  | 1,00 | - |
| Yes | 8 | 61,54 |  | 0,99 | 0,63 - 1,54 | | 2 | 15,38 |  | 0,75 | 0,20 – 2,80 | | 6 | 46,15 |  | 0,80 | 0,44 - 1,47 |
| **Had STI** |  |  | 0,712 |  |  | |  |  | 0,354^¶^ |  |  | |  |  | 0,409 |  |  |
| No | 89 | 61,81 |  | 1,00 | - | | 28 | 19,44 |  | 1,00 | - | | 80 | 55,56 |  | 1,00 | - |
| Yes | 10 | 66,67 |  | 1,08 | 0,74 - 1,58 | | 4 | 26,67 |  | 1,37 | 0,55 – 3,40 | | 10 | 66,67 |  | 1,20 | 0,81 - 1,77 |
| **STI Treatment** |  |  | 0,459 |  |  | |  |  | 0,409 |  |  | |  |  | 0,241 |  |  |
| No | 10 | 71,43 |  | 1,00 | - | | 4 | 28,57 |  | 1,00 | - | | 10 | 71,43 |  | 1,00 | - |
| Yes | 89 | 61,38 |  | 0,86 | 0,60 - 1,23 | | 28 | 19,31 |  | 0,68 | 0,28 – 1,65 | | 80 | 55,17 |  | 0,77 | 0,54 - 1,11 |
| **Ectopic pregnancy** |  |  | 0,592^¶^ |  |  | |  |  | 0,348^¶^ |  |  | |  |  | 0,527^¶^ |  |  |
| No | 95 | 62,09 |  | 1,00 | - | | 30 | 19,61 |  | 1,00 | - | | 87 | 56,86 |  | 1,00 | - |
| Yes | 4 | 66,67 |  | 1,07 | 0,60 - 1,92 | | 2 | 33,33 |  | 1,7 | 0,52 – 5,53 | | 3 | 50,00 |  | 0,88 | 0,39 - 1,99 |
| **Positive test for STI** |  |  | 0,592^¶^ |  |  | |  |  | 0,652^¶^ |  |  | |  |  | 0,473^¶^ |  |  |
| No | 95 | 62,09 |  | 1,00 | - | | 31 | 20,26 |  | 1,00 | - | | 86 | 56,21 |  | 1,00 | - |
| Yes | 4 | 66,67 |  | 1,07 | 0,60 - 1,92 | | 1 | 16,67 |  | 0,82 | 0,13 – 5,09 | | 4 | 66,67 |  | 1,19 | 0,66 - 2,13 |
| **Age of first sexual intercourse** |  |  | 0,176 |  |  | |  |  | **0,030** |  |  | |  |  | 0,117 |  |  |
| ≽ 15 years | 73 | 58,87 |  | 1,00 | - | | 20 | 16,13 |  | 1,00 | - | | 65 | 52,42 |  | 1,00 | - |
| Up to 14 years | 19 | 73,08 |  | 1,24 | 0,94 - 1,63 | | 9 | 34,62 |  | 2,15 | 1,10 – 4,18 | | 18 | 69,23 |  | 1,32 | 0,97 - 1,80 |
| **Had more than 1 partner during life** |  |  | 0,221 |  |  | |  |  | 0,580 |  |  | |  |  | 0,096 |  |  |
| No | 25 | 54,35 |  | 1,00 |  | | 8 | 17,39 |  | 1,00 | - | | 21 | 45,65 |  | 1,00 | - |
| Yes | 70 | 64,81 |  | 1,19 | 0,88 - 1,61 | | 23 | 21,30 |  | 1,22 | 0,59 – 2,54 | | 65 | 60,19 |  | 1,32 | 0,93 - 1,87 |
| **Pain during sexual intercourse** |  |  | 0,619 |  |  | |  |  | 0,835 |  |  | |  |  | 0,287 |  |  |
| No | 79 | 62,20 |  | 1,00 |  | | 25 | 19,69 |  | 1,00 | - | | 73 | 57,48 |  | 1,00 | - |
| Yes | 16 | 57,14 |  | 0,92 | 0,65 - 1,30 | | 6 | 21,43 |  | 1,09 | 0,49 – 2,41 | | 13 | 46,43 |  | 0,81 | 0,53 - 1,24 |
| **Bleeding during sexual intercourse** |  |  | 0,446^¶^ |  |  | |  |  | 0,575^¶^ |  |  | |  |  | 0,322^¶^ |  |  |
| No | 90 | 60,81 |  | 1,00 | - | | 30 | 20,27 |  | 1,00 | - | | 81 | 54,73 |  | 1,00 | - |
| Yes | 5 | 71,43 |  | 1,17 | 0,72 - 1,91 | | 1 | 14,29 |  | 0,70 | 0,11 – 4,48 | | 5 | 71,43 |  | 1,31 | 0,80 - 2,14 |
| **Condom use** |  |  | 0,644 |  |  | |  |  | 0,120 |  |  | |  |  | 0,657 |  |  |
| No / Sometimes | 73 | 60,33 |  | 1,00 | - | | 21 | 17,36 |  | 1,00 | - | | 66 | 54,55 |  | 1,00 | - |
| Yes | 22 | 64,71 |  | 1,07 | 0,80 - 1,43 | | 10 | 29,41 |  | 1,70 | 0,88 -3,25 | | 20 | 58,82 |  | 1,08 | 0,78 - 1,49 |
| **Time of last gynecological examination** |  |  | 0,701 |  |  | |  |  | 0,549 |  |  | |  |  | 0,808 |  |  |
| In the last year | 53 | 60,92 |  | 1,00 | - | | 16 | 18,39 |  | 1,00 | - | | 50 | 57,47 |  | 1,00 | - |
| More than 1 year | 46 | 63,89 |  | 1,05 | 0,82 - 1,34 | | 16 | 22,22 |  | 1,20 | 0,65 – 2,25 | | 40 | 55,56 |  | 0,97 | 0,73 - 1,27 |
| **Use of contraceptives** |  |  | 0,075 |  |  | |  |  | 0,605 |  |  | |  |  | 0,119 |  |  |
| No | 53 | 68,83 |  | 1,00 | - | | 17 | 22,08 |  | 1,00 | - | | 48 | 62,34 |  | 1,00 | - |
| Yes | 44 | 55,00 |  | 0,80 | 0,62 - 1,03 | | 15 | 18,75 |  | 0,85 | 0,46 – 1,58 | | 40 | 50,00 |  | 0,80 | 0,61 - 1,06 |
| **Number of pregnancies** |  |  | 0,891 |  |  | |  |  | 0,444 |  |  | |  |  | 0,858 |  |  |
| 1 | 33 | 64,71 |  | 1,00 | - | | 13 | 25,49 |  | 1,00 | - | | 30 | 58,82 |  | 1,00 | - |
| 2 | 28 | 62,22 |  | 0,96 | 0,71 - 1,30 | | 9 | 20,00 |  | 0,78 | 0,37 – 1,66 | | 24 | 53,33 |  | 0,91 | 0,63 - 1,30 |
| 3 or more | 38 | 60,32 |  | 0,93 | 0,70 - 1,24 | | 10 | 15,87 |  | 0,62 | 0,30 – 1,30 | | 36 | 57,14 |  | 0,97 | 0,71 - 1,33 |
| **Abortion** |  |  | 0,848 |  |  | |  |  | 0,766 |  |  | |  |  | 0,748 |  |  |
| None | 70 | 62,50 |  | 1,00 | - | | 22 | 19,64 |  | 1,00 | - | | 64 | 57,14 |  | 1,00 | - |
| 1 or more | 28 | 60,87 |  | 0,97 | 0,74 - 1,28 | | 10 | 21,74 |  | 1,11 | 0,57 – 2,15 | | 25 | 54,35 |  | 0,95 | 0,70 - 1,30 |
| **Premature birth** |  |  | 0,502 |  |  | |  |  | 0,295 |  |  | |  |  | 0,967 |  |  |
| None | 81 | 63,28 |  | 1,00 | - | | 28 | 21,88 |  | 1,00 | - | | 72 | 56,25 |  | 1,00 | - |
| 1 or more | 17 | 56,67 |  | 0,89 | 0,64 - 1,26 | | 4 | 13,33 |  | 0,61 | 0,23 – 1,61 | | 17 | 56,67 |  | 1,01 | 0,71 - 1,43 |
| **Stillborn** |  |  | 0,978 |  |  | |  |  | 0,541 |  |  | |  |  | 0,854 |  |  |
| None | 81 | 61,83 |  | 1,00 | - | | 27 | 20,61 |  | 1,00 | - | | 73 | 55,73 |  | 1,00 | - |
| 1 or more | 16 | 61,54 |  | 0,99 | 0,71 - 1,39 | | 4 | 15,38 |  | 0,75 | 0,28 – 1,96 | | 15 | 57,69 |  | 1,04 | 0,72 - 1,49 |
| **Expected prenatal consultations** |  |  | 0,578 |  |  | |  |  | 0,323 |  |  | |  |  | 0,556 |  |  |
| Greater than or equal to 6 | 72 | 61,54 |  | 1,00 | - | | 25 | 21,37 |  | 1,00 | - | | 65 | 55,56 |  | 1,00 | - |
| Less than 6 | 24 | 66,67 |  | 1,08 | 0,82 - 1,42 | | 5 | 13,89 |  | 0,65 | 0,27 – 1,58 | | 22 | 61,11 |  | 1,1 | 0,81 - 1,50 |
| **Antibiotic use in the last 3 months** |  |  | 0,395 |  |  | |  |  | 0,146 |  |  | |  |  | 0,450 |  |  |
| No | 66 | 64,71 |  | 1,00 | - | | 17 | 16,67 |  | 1,00 | - | | 60 | 58,82 |  | 1,00 | - |
| Yes | 33 | 57,89 |  | 0,89 | 0,69 - 1,17 | | 15 | 26,32 |  | 1,58 | 0,85 – 2,92 | | 30 | 52,63 |  | 0,89 | 0,67 - 1,20 |
| **Presence of symptoms** |  |  | 0,765 |  |  | |  |  | 0,909 |  |  | |  |  | 0,614 |  |  |
| None | 18 | 62,07 |  | 1,00 | - | | 5 | 17,24 |  | 1,00 | - | | 17 | 58,62 |  | 1,00 | - |
| 1 | 32 | 58,18 |  | 0,94 | 0,65 - 1,35 | | 11 | 20,00 |  | 1,16 | 0,44 – 3,03 | | 28 | 50,91 |  | 0,87 | 0,58 - 1,30 |
| 2 or more | 49 | 64,47 |  | 1,04 | 0,75 - 1,45 | | 16 | 21,05 |  | 1,22 | 0,49 – 3,04 | | 45 | 59,21 |  | 1,01 | 0,71 - 1,45 |
| **Vaginal discharge** |  |  | 0,665 |  |  | |  |  | 0,780 |  |  | |  |  | 0,995 |  |  |
| No | 46 | 60,53 |  | 1,00 | - | | 16 | 21,05 |  | 1,00 | - | | 43 | 56,58 |  | 1,00 | - |
| Yes | 53 | 63,86 |  | 1,06 | 0,83 - 1,35 | | 16 | 19,28 |  | 0,92 | 0,49 – 1,70 | | 47 | 56,63 |  | 1,00 | 0,76 - 1,32 |
| **Itching** |  |  | 0,435 |  |  | |  |  | 0,512^¶^ |  |  | |  |  | 0,367 |  |  |
| No | 83 | 61,03 |  | 1,00 | - | | 27 | 19,85 |  | 1,00 | - | | 75 | 55,15 |  | 1,00 | - |
| Yes | 16 | 69,57 |  | 1,14 | 0,84 - 1,54 | | 5 | 21,74 |  | 1,10 | 0,47 – 2,56 | | 15 | 65,22 |  | 1,18 | 0,85 - 1,65 |
| **Vaginal erythema** |  |  | 0,626^¶^ |  |  | |  |  | 0,736^¶^ |  |  | |  |  | 0,624^¶^ |  |  |
| No | 96 | 62,34 |  | 1,00 | - | | 31 | 20,13 |  | 1,00 | - | | 87 | 56,49 |  | 1,00 | - |
| Yes | 3 | 60,00 |  | 0,96 | 0,46 - 1,99 | | 1 | 20,00 |  | 0,99 | 0,17 – 5,93 | | 3 | 60,00 |  | 1,06 | 0,51 - 2,21 |
| **Pelvic pain** |  |  | 0,921 |  |  | |  |  | 0,970 |  |  | |  |  | 0,620 |  |  |
| No | 52 | 61,90 |  | 1,00 | - | | 17 | 20,24 |  | 1,00 | - | | 46 | 54,76 |  | 1,00 | - |
| Yes | 47 | 62,67 |  | 1,01 | 0,79 - 1,29 | | 15 | 20,00 |  | 0,99 | 0,53 – 1,84 | | 44 | 58,67 |  | 1,07 | 0,82 - 1,41 |
| **Genital vesicles** |  |  | 0,319^¶^ |  |  | |  |  | 0,507^¶^ |  |  | |  |  | 0,401^¶^ |  |  |
| No | 98 | 62,82 |  | 1,00 | - | | 32 | 20,51 |  | 1,00 | - | | 89 | 57,05 |  | 1,00 | - |
| Yes | 1 | 33,33 |  | 0,53 | 0,11 - 2,65 | | ** | ** | ** | ** | ** | | 1 | 33,33 |  | 0,58 | 0,12 - 2,93 |
| **Pain when urinating** |  |  | 0,599 |  |  | |  |  | 0,682 |  |  | |  |  | 0,756 |  |  |
| No | 84 | 63,16 |  | 1,00 | - | | 26 | 19,55 |  | 1,00 | - | | 76 | 57,14 |  | 1,00 | - |
| Yes | 15 | 57,69 |  | 0,91 | 0,64 - 1,30 | | 6 | 23,08 |  | 1,18 | 0,54 – 2,59 | | 14 | 53,85 |  | 0,94 | 0,64 - 1,39 |
| **Swollen glands in the groin** |  |  | 0,312 |  |  | |  |  | 0,258^¶^ |  |  | |  |  | 0,180 |  |  |
| No | 92 | 60,93 |  | 1,00 | - | | 29 | 19,21 |  | 1,00 | - | | 83 | 54,97 |  | 1,00 | - |
| Yes | 7 | 77,78 |  | 1,28 | 0,88 - 1,85 | | 3 | 33,33 |  | 1,74 | 0,65 – 4,64 | | 7 | 77,78 |  | 1,41 | 0,97 - 2,07 |
| **Other gynecological changes** |  |  | 0,296 |  |  | |  |  | 0,633 |  |  | |  |  | 0,155 |  |  |
| No | 80 | 64,00 |  | 1,00 | - | | 24 | 19,20 |  | 1,00 | - | | 74 | 59,20 |  | 1,00 | - |
| Yes | 19 | 54,29 |  | 0,85 | 0,61 - 1,18 | | 8 | 22,86 |  | 1,19 | 0,59 – 2,42 | | 16 | 45,71 |  | 0,77 | 0,52 - 1,14 |
| **Partner treatment for infertility** |  |  | 0,034 |  |  | |  |  | 0,495^¶^ |  |  | |  |  | **0,000** |  |  |
| No | 95 | 63,76 |  | 1,00 | - | | 29 | 20,42 |  | 1,00 | - | | 87 | 61,27 |  | 1,00 | - |
| Yes | 3 | 30,00 |  | 0,47 | 0,18 - 1,23 | | 3 | 16,67 |  | 0,82 | 0,28 – 2,42 | | 3 | 16,67 |  | 0,27 | 0,10 - 0,77 |
| **Partner treatment for STIs** |  |  | 0,316 |  |  | |  |  | 0,083^¶^ |  |  | |  |  | 0,520 |  |  |
| No | 91 | 61,07 |  | 1,00 | - | | 28 | 18,79 |  | 1,00 | - | | 83 | 55,70 |  | 1,00 | - |
| Yes | 7 | 77,78 |  | 1,27 | 0,88 - 1,85 | | 4 | 44,44 |  | 2,37 | 1,06 – 5,30 | | 6 | 66,67 |  | 1,20 | 0,74 - 1,94 |

*n = Absolute frequency; †P = Prevalence; ‡PR = Crude prevalence ratio; §CI95%: Confidence interval 95%; ¶: Fisher's exact test; **Statistical analysis was not possible

**Table S2.** Bivariate analysis for the prevalence of *Ureaplasma spp.*, *Ureaplasma parvum* and *Ureaplasma urealyticum* in pregnant women receiving high-risk prenatal care in southwestern Bahia (n = 164). Brazil, 2021-2022.

|  | *Ureaplasma* spp. | | | | | *Ureaplasma urealyticum* | | | | | | *Ureaplasma parvum* | | | | | |
| --- | --- | --- | --- | --- | --- | --- | --- | --- | --- | --- | --- | --- | --- | --- | --- | --- | --- |
| Variables | n* | P(%)† | p-value | PR‡ | CI 95%§ | | n* | P(%)† | p-value | PR‡ | CI 95%§ | | n* | P(%) | p-value | PR‡ | CI 95%§ |
| **Age** |  |  | 0,237 |  |  | |  |  | 0,093 |  |  | |  |  | 0,187 |  |  |
| Up to 19 years old | 16 | 72,73 |  | 1,00 | - | | 4 | 18,18 |  | 1,00 | - | | 15 | 68,18 |  | 1,00 | - |
| 20 - 34 | 48 | 52,75 |  | 0,73 | 0,90 - 1,85 | | 6 | 6,59 |  | 1,05 | 0,35 – 3,11 | | 44 | 48,35 |  | 1,49 | 0,97 – 2,29 |
| 35 or more | 26 | 56,52 |  | 0,78 | 0,68 - 1,29 | | 8 | 17,39 |  | 0,38 | 0,14 – 1,03 | | 21 | 45,65 |  | 1,06 | 0,72 – 1,55 |
| **Marital status** |  |  | 0,241 |  |  | |  |  | 0,172^¶^ |  |  | |  |  | 0,098 |  |  |
| With partner | 80 | 55,17 |  | 1,00 | - | | 18 | 12,41 |  | 1,00 | - | | 70 | 48,28 |  | 1,00 | - |
| Without partner | 10 | 71,43 |  | 1,29 | 0,90 - 1,86 | | ** | ** |  | ** | ** | | 10 | 71,43 |  | 1,48 | 1,02 – 2,15 |
| **Origin** |  |  | 0,330 |  |  | |  |  | 0,761 |  |  | |  |  | 0,437 |  |  |
| Rural area | 20 | 50,00 |  | 1,00 | - | | 4 | 10,00 |  | 1,00 | - | | 18 | 45,00 |  | 1,00 | - |
| Urban area | 70 | 58,82 |  | 1,18 | 0,83 - 1,66 | | 14 | 11,76 |  | 1,17 | 0,41 – 3,38 | | 62 | 52,10 |  | 1,16 | 0,79 – 1,70 |
| **Color** |  |  | 0,484 |  |  | |  |  | 0,236 |  |  | |  |  | 0,815 |  |  |
| Non-black | 18 | 51,43 |  | 1,00 | - | | 2 | 5,71 |  | 1,00 | - | | 17 | 48,57 |  | 1,00 | - |
| Black | 72 | 58,06 |  | 1,13 | 0,79 - 1,61 | | 16 | 12,90 |  | 2,26 | 0,54 – 9,40 | | 63 | 50,81 |  | 1,05 | 0,71 – 1,54 |
| **Education** |  |  | 0,217 |  |  | |  |  | 0,744 |  |  | |  |  | 0,066 |  |  |
| Up to 11 years | 33 | 50,77 |  | 1,00 | - | | 8 | 12,31 |  | 1,00 | - | | 27 | 41,54 |  | 1,00 | - |
| 12 or more | 57 | 60,64 |  | 1,19 | 0,89 - 1,60 | | 10 | 10,64 |  | 0,86 | 0,36 – 2,08 | | 53 | 56,38 |  | 1,36 | 0,97 – 1,91 |
| **Income** |  |  | 0,072 |  |  | |  |  | 0,508 |  |  | |  |  | 0,206 |  |  |
| Less than 1 minimum wage | 54 | 63,53 |  | 1,00 | - | | 11 | 12,94 |  | 1,00 | - | | 47 | 55,29 |  | 1,00 | - |
| 1 minimum wage or more | 36 | 49,32 |  | 0,78 | 0,58 - 1,03 | | 7 | 9,59 |  | 0,74 | 0,30 – 1,82 | | 33 | 45,21 |  | 0,82 | 0,60 – 1,12 |
| **Gestational age** |  |  | 0,356 |  |  | |  |  | 0,051 |  |  | |  |  | 0,967 |  |  |
| 1st trimester | 10 | 71,43 |  | 1,00 | - | | 4 | 28,57 |  | 1,00 | - | | 7 | 50,00 |  | 1,00 | - |
| 2nt trimester | 39 | 59,09 |  | 0,83 | 0,56 - 1,22 | | 8 | 12,12 |  | 0,42 | 0,15 – 1,22 | | 34 | 51,52 |  | 1,03 | 0,58 – 1,83 |
| 3rd trimester | 39 | 52,00 |  | 0,73 | 0,49 - 1,08 | | 5 | 6,67 |  | 0,23 | 0,07 - 0,77 | | 37 | 49,33 |  | 0,99 | 0,56 – 1,75 |
| **Physical activity** |  |  | 0,282 |  |  | |  |  | 0,156^¶^ |  |  | |  |  | 0,730 |  |  |
| No | 68 | 54,40 |  | 1,00 | - | | 12 | 9,60 |  | 1,00 | - | | 62 | 49,60 |  | 1,00 | - |
| Yes | 22 | 64,71 |  | 1,19 | 0,88 - 1,60 | | 6 | 17,65 |  | 1,84 | 0,74 – 4,55 | | 18 | 52,94 |  | 1,07 | 0,74 – 1,54 |
| **Alcoholic beverage** |  |  | 0,531 |  |  | |  |  | 0,730^¶^ |  |  | |  |  | 0,508^¶^ |  |  |
| No | 84 | 56,00 |  | 1,00 | - | | 17 | 11,33 |  | 1,00 | - | | 75 | 50,00 |  | 1,00 | - |
| Yes | 6 | 66,67 |  | 1,19 | 0,73 - 1,93 | | 1 | 11,11 |  | 0,98 | 0,15 – 6,60 | | 5 | 55,56 |  | 1,11 | 0,61 – 2,04 |
| **Myoma** |  |  | 0,104 |  |  | |  |  | 0,433^¶^ |  |  | |  |  | 0,108 |  |  |
| No | 84 | 58,74 |  | 1,00 | - | | 17 | 11,89 |  | 1,00 | - | | 75 | 52,45 |  | 1,00 | - |
| Yes | 6 | 37,5 |  | 0,64 | 0,33 - 1,22 | | 1 | 6,25 |  | 0,53 | 0,07 – 3,72 | | 5 | 31,25 |  | 0,60 | 0,28 – 1,26 |
| **Polycystic ovaries** |  |  | **0,022** |  |  | |  |  | 0,498^¶^ |  |  | |  |  | 0,039 |  |  |
| No | 82 | 60,29 |  | 1,00 |  | | 16 | 11,76 |  | 1,00 |  | | 73 | 53,68 |  | 1,00 |  |
| Yes | 8 | 34,78 |  | 0,58 | 0,32 - 1,03 | | 2 | 8,70 |  | 0,74 | 0,18 – 3,02 | | 7 | 30,43 |  | 0,58 | 0,30 – 1,07 |
| **Other gynecological changes** |  |  | 0,428 |  |  | |  |  | 0,448^¶^ |  |  | |  |  | 0,372 |  |  |
| No | 84 | 57,53 |  | 1,00 | - | | 16 | 10,96 |  | 1,00 | - | | 75 | 51,37 |  | 1,00 | - |
| Yes | 6 | 46,15 |  | 0,80 | 0,44 - 1,47 | | 2 | 15,38 |  | 1,40 | 0,36 – 5,47 | | 5 | 38,46 |  | 0,75 | 0,37 – 1,52 |
| **Had STI** |  |  | 0,409 |  |  | |  |  | 0,071^¶^ |  |  | |  |  | 0,430 |  |  |
| No | 80 | 55,56 |  | 1,00 | - | | 14 | 9,72 |  | 1,00 | - | | 71 | 49,31 |  | 1,00 | - |
| Yes | 10 | 66,67 |  | 1,20 | 0,81 - 1,77 | | 4 | 26,67 |  | 2,74 | 1,03 – 7,30 | | 9 | 60,00 |  | 1,22 | 0,78 – 1,90 |
| **STI Treatment** |  |  | 0,241 |  |  | |  |  | **0,033** |  |  | |  |  | 0,274 |  |  |
| No | 10 | 71,43 |  | 1,00 | - | | 4 | 28,57 |  | 1,00 | - | | 9 | 64,29 |  | 1,00 | - |
| Yes | 80 | 55,17 |  | 0,77 | 0,54 - 1,11 | | 14 | 9,66 |  | 0,34 | 0,13 – 0,89 | | 71 | 48,97 |  | 0,76 | 0,50 – 1,17 |
| **Ectopic pregnancy** |  |  | 0,527^¶^ |  |  | |  |  | **0,020**^¶^ |  |  | |  |  | **0,014**^¶^ |  |  |
| No | 87 | 56,86 |  | 1,00 | - | | 15 | 9,80 |  | 1,00 | - | | 80 | 52,29 |  | 1,00 | - |
| Yes | 3 | 50,00 |  | 0,88 | 0,39 - 1,99 | | 3 | 50,00 |  | 5,1 | 2,00 – 13,00 | | ** | ** |  | ** | ** |
| **Positive test for STI** |  |  | 0,473^¶^ |  |  | |  |  | 0,138^¶^ |  |  | |  |  | 0,347^¶^ |  |  |
| No | 86 | 56,21 |  | 1,00 | - | | 16 | 10,46 |  | 1,00 | - | | 76 | 49,67 |  | 1,00 | - |
| Yes | 4 | 66,67 |  | 1,19 | 0,66 - 2,13 | | 2 | 33,33 |  | 3,18 | 0,93 – 10,87 | | 4 | 66,67 |  | 1,34 | 0,74 – 2,42 |
| **Age of first sexual intercourse** |  |  | 0,117 |  |  | |  |  | 0,335^¶^ |  |  | |  |  | 0,149 |  |  |
| ≽ 15 years | 65 | 52,42 |  | 1,00 | - | | 13 | 10,48 |  | 1,00 | - | | 57 | 45,97 |  | 1,00 | - |
| Up to 14 years | 18 | 69,23 |  | 1,32 | 0,97 - 1,80 | | 4 | 15,38 |  | 1,47 | 0,52 – 4,16 | | 16 | 61,54 |  | 1,34 | 0,93 -1,92 |
| **Had more than 1 partner during life** |  |  | 0,096 |  |  | |  |  | 0,451 |  |  | |  |  | 0,192 |  |  |
| No | 21 | 45,65 |  | 1,00 | - | | 4 | 8,70 |  | 1,00 | - | | 19 | 41,30 |  | 1,00 | - |
| Yes | 65 | 60,19 |  | 1,32 | 0,93 - 1,87 | | 14 | 12,96 |  | 1,49 | 0,52 – 4,30 | | 57 | 52,78 |  | 1,28 | 0,87 – 1,89 |
| **Pain during sexual intercourse** |  |  | 0,287 |  |  | |  |  | 0,329^¶^ |  |  | |  |  | 0,254 |  |  |
| No | 73 | 57,48 |  | 1,00 | - | | 16 | 12,60 |  | 1,00 | - | | 65 | 51,18 |  | 1,00 | - |
| Yes | 13 | 46,43 |  | 0,81 | 0,53 - 1,24 | | 2 | 7,14 |  | 0,57 | 0,14 – 2,34 | | 11 | 39,29 |  | 0,77 | 0,47 – 1,26 |
| **Bleeding during sexual intercourse** |  |  | 0,322^¶^ |  |  | |  |  | 0,586 |  |  | |  |  | 0,478^¶^ |  |  |
| No | 81 | 54,73 |  | 1,00 | - | | 17 | 11,49 |  | 1,00 | - | | 72 | 48,65 |  | 1,00 | - |
| Yes | 5 | 71,43 |  | 1,31 | 0,80 - 2,14 | | 1 | 14,29 |  | 1,24 | 0,19 – 8,11 | | 4 | 57,14 |  | 1,17 | 0,60 – 2,28 |
| **Condom use** |  |  | 0,657 |  |  | |  |  | 0,172^¶^ |  |  | |  |  | 0,794 |  |  |
| No / Sometimes | 66 | 54,55 |  | 1,00 | - | | 12 | 9,92 |  | 1,00 | - | | 60 | 49,59 |  | 1,00 | - |
| Yes | 20 | 58,82 |  | 1,08 | 0,78 - 1,49 | | 6 | 17,65 |  | 1,78 | 0,72 – 4,40 | | 16 | 47,06 |  | 0,95 | 0,64 – 1,42 |
| **Time of last gynecological examination** |  |  | 0,808 |  |  | |  |  | 0,939 |  |  | |  |  | 0,942 |  |  |
| In the last year | 50 | 57,47 |  | 1,00 | - | | 10 | 11,49 |  | 1,00 | - | | 44 | 50,57 |  | 1,00 | - |
| More than 1 year | 40 | 55,56 |  | 0,97 | 0,73 - 1,27 | | 8 | 11,11 |  | 0,97 | 0,40 – 2,37 | | 36 | 50,00 |  | 0,99 | 0,72 – 1,35 |
| **Use of contraceptives** |  |  | 0,119 |  |  | |  |  | 0,678 |  |  | |  |  | **0,031** |  |  |
| No | 48 | 62,34 |  | 1,00 | - | | 8 | 10,39 |  | 1,00 | - | | 45 | 58,44 |  | 1,00 | - |
| Yes | 40 | 50,00 |  | 0,80 | 0,61 - 1,06 | | 10 | 12,50 |  | 1,20 | 0,50 – 2,90 | | 33 | 41,25 |  | 0,71 | 0,51 – 0,98 |
| **Number of pregnancies** |  |  | 0,858 |  |  | |  |  | 0,860 |  |  | |  |  | 0,262 |  |  |
| 1 | 30 | 58,82 |  | 1,00 | - | | 5 | 9,80 |  | 1,00 | - | | 28 | 54,90 |  | 1,00 | - |
| 2 | 24 | 53,33 |  | 0,91 | 0,63 - 1,30 | | 6 | 13,33 |  | 1,36 | 0,44 – 4,17 | | 18 | 40,00 |  | 0,73 | 0,47 – 1,13 |
| 3 or more | 36 | 57,14 |  | 0,97 | 0,71 - 1,33 | | 7 | 11,11 |  | 1,13 | 0,38 – 3,38 | | 34 | 53,97 |  | 0,98 | 0,70 – 1,38 |
| **Abortion** |  |  | 0,748 |  |  | |  |  | 0,494 |  |  | |  |  | 1,000 |  |  |
| None | 64 | 57,14 |  | 1,00 | - | | 14 | 12,50 |  | 1,00 | - | | 56 | 50,00 |  | 1,00 | - |
| 1 or more | 25 | 54,35 |  | 0,95 | 0,70 - 1,30 | | 4 | 8,70 |  | 0,70 | 0,24 – 2,00 | | 23 | 50,00 |  | 1,00 | 0,71 – 1,41 |
| **Premature birth** |  |  | 0,967 |  |  | |  |  | 0,542^¶^ |  |  | |  |  | 0,685 |  |  |
| None | 72 | 56,25 |  | 1,00 | - | | 15 | 11,72 |  | 1,00 | - | | 65 | 50,78 |  | 1,00 | - |
| 1 or more | 17 | 56,67 |  | 1,01 | 0,71 - 1,43 | | 3 | 10,00 |  | 0,85 | 0,26 – 2,77 | | 14 | 46,67 |  | 0,92 | 0,60 – 1,40 |
| **Stillborn** |  |  | 0,854 |  |  | |  |  | 0,395^¶^ |  |  | |  |  | 0,642 |  |  |
| None | 73 | 55,73 |  | 1,00 | - | | 16 | 12,21 |  | 1,00 | - | | 64 | 48,85 |  | 1,00 | - |
| 1 or more | 15 | 57,69 |  | 1,04 | 0,72 - 1,49 | | 2 | 7,69 |  | 0,63 | 0,15 – 2,59 | | 14 | 53,85 |  | 1,10 | 0,74 – 1,64 |
| **Expected prenatal consultations** |  |  | 0,556 |  |  | |  |  | 0,367^¶^ |  |  | |  |  | 0,473 |  |  |
| Greater than or equal to 6 | 65 | 55,56 |  | 1,00 | - | | 12 | 10,26 |  | 1,00 | - | | 57 | 48,72 |  | 1,00 | - |
| Less than 6 | 22 | 61,11 |  | 1,1 | 0,81 - 1,50 | | 5 | 13,89 |  | 1,35 | 0,51 – 3,60 | | 20 | 55,56 |  | 1,14 | 0,81 – 1,61 |
| **Antibiotic use in the last 3 months** |  |  | 0,450 |  |  | |  |  | 0,448 |  |  | |  |  | 0,662 |  |  |
| No | 60 | 58,82 |  | 1,00 | - | | 13 | 12,75 |  | 1,00 | - | | 50 | 49,02 |  | 1,00 | - |
| Yes | 30 | 52,63 |  | 0,89 | 0,67 - 1,20 | | 5 | 8,77 |  | 0,69 | 0,26 – 1,84 | | 30 | 52,63 |  | 1,07 | 0,78 – 1,47 |
| **Presence of symptoms** |  |  | 0,614 |  |  | |  |  | 0,625 |  |  | |  |  | 0,497 |  |  |
| None | 17 | 58,62 |  | 1,00 | - | | 3 | 10,34 |  | 1,00 | - | | 15 | 51,72 |  | 1,00 | - |
| 1 | 28 | 50,91 |  | 0,87 | 0,58 - 1,30 | | 8 | 14,55 |  | 1,41 | 0,40 – 4,92 | | 24 | 43,64 |  | 0,84 | 0,53 – 1,34 |
| 2 or more | 45 | 59,21 |  | 1,01 | 0,71 - 1,45 | | 7 | 9,21 |  | 0,89 | 0,25 – 3,23 | | 41 | 53,95 |  | 1,04 | 0,69 – 1,57 |
| **Vaginal discharge** |  |  | 0,995 |  |  | |  |  | **0,028** |  |  | |  |  | 0,940 |  |  |
| No | 43 | 56,58 |  | 1,00 | - | | 13 | 17,11 |  | 1,00 | - | | 38 | 50,00 |  | 1,00 | - |
| Yes | 47 | 56,63 |  | 1,00 | 0,76 - 1,32 | | 5 | 6,02 |  | 0,35 | 0,13 – 0,94 | | 42 | 50,60 |  | 1,01 | 0,74 – 1,38 |
| **Itching** |  |  | 0,367 |  |  | |  |  | 0,249^¶^ |  |  | |  |  | 0,274 |  |  |
| No | 75 | 55,15 |  | 1,00 | - | | 14 | 10,29 |  | 1,00 | - | | 66 | 48,53 |  | 1,00 | - |
| Yes | 15 | 65,22 |  | 1,18 | 0,85 - 1,65 | | 4 | 17,39 |  | 1,69 | 0,61 – 4,70 | | 14 | 60,87 |  | 1,25 | 0,86 – 1,82 |
| **Vaginal erythema** |  |  | 0,624^¶^ |  |  | |  |  | 0,544^¶^ |  |  | |  |  | 0,506^¶^ |  |  |
| No | 87 | 56,49 |  | 1,00 | - | | 18 | 11,69 |  | 1,00 | - | | 77 | 50,00 |  | 1,00 | - |
| Yes | 3 | 60,00 |  | 1,06 | 0,51 - 2,21 | | ** | ** |  | ** | ** | | 3 | 60,00 |  | 1,20 | 0,57 – 2,50 |
| **Pelvic pain** |  |  | 0,620 |  |  | |  |  | 0,449 |  |  | |  |  | 0,933 |  |  |
| No | 46 | 54,76 |  | 1,00 | - | | 8 | 9,52 |  | 1,00 | - | | 42 | 50,00 |  | 1,00 | - |
| Yes | 44 | 58,67 |  | 1,07 | 0,82 - 1,41 | | 10 | 13,33 |  | 1,4 | 0,58 – 3,37 | | 38 | 50,67 |  | 1,01 | 0,74 – 1,38 |
| **Genital vesicles** |  |  | 0,401^¶^ |  |  | |  |  | 0,696^¶^ |  |  | |  |  | 0,495^¶^ |  |  |
| No | 89 | 57,05 |  | 1,00 | - | | 18 | 11,54 |  | 1,00 | - | | 79 | 50,64 |  | 1,00 | - |
| Yes | 1 | 33,33 |  | 0,58 | 0,12 - 2,93 | | ** | ** |  | ** | ** | | 1 | 33,33 |  | 0,66 | 0,13 – 3,30 |
| **Pain when urinating** |  |  | 0,756 |  |  | |  |  | 0,335^¶^ |  |  | |  |  | 0,972 |  |  |
| No | 76 | 57,14 |  | 1,00 | - | | 14 | 10,53 |  | 1,00 | - | | 67 | 50,38 |  | 1,00 | - |
| Yes | 14 | 53,85 |  | 0,94 | 0,64 - 1,39 | | 4 | 15,38 |  | 1,46 | 0,52 – 4,10 | | 13 | 50,00 |  | 0,99 | 0,65 – 1,51 |
| **Swollen glands in the groin** |  |  | 0,180 |  |  | |  |  | 0,066^¶^ |  |  | |  |  | 0,248^¶^ |  |  |
| No | 83 | 54,97 |  | 1,00 | - | | 15 | 9,93 |  | 1,00 | - | | 74 | 49,01 |  | 1,00 | - |
| Yes | 7 | 77,78 |  | 1,41 | 0,97 - 2,07 | | 3 | 33,33 |  | 3,36 | 1,18 – 9,54 | | 6 | 66,67 |  | 1,36 | 0,83 – 2,22 |
| **Other gynecological changes** |  |  | 0,155 |  |  | |  |  | 0,578^¶^ |  |  | |  |  | 0,339 |  |  |
| No | 74 | 59,20 |  | 1,00 | - | | 14 | 11,20 |  | 1,00 | - | | 65 | 52,00 |  | 1,00 | - |
| Yes | 16 | 45,71 |  | 0,77 | 0,52 - 1,14 | | 4 | 11,43 |  | 1,02 | 0,36 – 2,91 | | 15 | 42,86 |  | 0,82 | 0,54 – 1,25 |
| **Partner treatment for infertility** |  |  | **0,000** |  |  | |  |  | 0,102^¶^ |  |  | |  |  | **0,003** |  |  |
| No | 87 | 61,27 |  | 1,00 | - | | 18 | 12,68 |  | 1,00 | - | | 77 | 54,23 |  | 1,00 | - |
| Yes | 3 | 16,67 |  | 0,27 | 0,10 - 0,77 | | ** | ** |  | ** | ** | | 3 | 16,67 |  | 0,31 | 0,11 – 0,88 |
| **Partner treatment for STIs** |  |  | 0,520 |  |  | |  |  | 0,273^¶^ |  |  | |  |  | 0,500^¶^ |  |  |
| No | 83 | 55,70 |  | 1,00 | - | | 16 | 10,74 |  | 1,00 | - | | 74 | 49,66 |  | 1,00 | - |
| Yes | 6 | 66,67 |  | 1,20 | 0,74 - 1,94 | | 2 | 22,22 |  | 2,07 | 0,56 – 7,68 | | 5 | 55,56 |  | 1,12 | 0,61 – 2,06 |

*n = Absolute frequency; †P = Prevalence; ‡PR = Crude prevalence ratio; §CI95%: Confidence interval 95%; ¶: Fisher's exact test; **Statistical analysis was not possible

**Table S3.** Bivariate analysis for the prevalence of *Mycoplasma spp.*, *Mycoplasma genitalium* and *Mycoplasma hominis* in pregnant women receiving high-risk prenatal care in southwestern Bahia (n = 164). Brazil, 2021-2022.

|  | *Mycoplasma* spp. | | | | | *Mycoplasma genitalium* | | | | | | *Mycoplasma hominis* | | | | |
| --- | --- | --- | --- | --- | --- | --- | --- | --- | --- | --- | --- | --- | --- | --- | --- | --- |
| Variables | n* | P(%)† | p-value | PR‡ | CI 95%§ | | n* | P(%)† | p-value | PR‡ | CI95%§ | n* | P(%)† | p-value | PR‡ | CI 95%§ |
| **Age** |  |  | **0,001** |  |  | |  |  | 0,341^¶^ |  |  |  |  | **0,000** |  |  |
| Up to 19 years old | 11 | 50,00 |  | 1,00 | - | | 1 | 4,55 |  | 1,00 | - | 11 | 50,00 |  | 1,00 | - |
| 20 - 34 | 13 | 14,29 |  | 2,88 | 1,35 – 6,14 | | 1 | 1,10 |  | ** | ** | 12 | 13,19 |  | 2,88 | 1,35 – 6,14 |
| 35 or more | 8 | 17,39 |  | 0,82 | 0,37 – 1,84 | | ** | ** |  | ** | ** | 8 | 17,39 |  | 0,76 | 0,33 – 1,73 |
| **Marital status** |  |  | 0,037^¶^ |  |  | |  |  | 0,831^¶^ |  |  |  |  | 0,041^¶^ |  |  |
| With partner | 32 | 22,07 |  | 1,00 | - | | 2 | 1,38 |  | 1,00 | - | 31 | 21,38 |  | 1,00 | - |
| Without partner | ** | ** |  | ** | ** | | ** | ** |  | ** | ** | ** | ** |  | ** | ** |
| **Origin** |  |  | 0,374 |  |  | |  |  | 0,559^¶^ |  |  |  |  | 0,310 |  |  |
| Rural area | 10 | 25,00 |  | 1,00 | - | | ** | ** |  | ** | ** | 10 | 25,00 |  | 1,00 | - |
| Urban area | 22 | 18,49 |  | 0,74 | 0,38 – 1,43 | | 2 | 1,68 |  | - | - | 21 | 17,65 |  | 0,71 | 0,36 – 1,37 |
| **Color** |  |  | 0,329 |  |  | |  |  | 0,607^¶^ |  |  |  |  | 0,378 |  |  |
| Non-black | 5 | 14,29 |  | 1,00 | - | | ** | ** |  | ** | ** | 5 | 14,29 |  | 1,00 | - |
| Black | 27 | 21,77 |  | 1,52 | 0,63 – 3,68 | | 2 | 1,61 |  | - | - | 26 | 20,97 |  | 1,47 | 0,61 – 3,55 |
| **Education** |  |  | 0,663 |  |  | |  |  | 0,166^¶^ |  |  |  |  | 0,496 |  |  |
| Up to 11 years | 12 | 18,46 |  | 1,00 | - | | 2 | 3,08 |  | 1,00 | - | 11 | 16,92 |  | 1,00 | - |
| 12 or more | 20 | 21,28 |  | 1,15 | 0,61 – 2,19 | | ** | ** |  | ** | ** | 20 | 21,28 |  | 1,26 | 0,65 – 2,45 |
| **Income** |  |  | 0,629 |  |  | |  |  | 0,288^¶^ |  |  |  |  | 0,500 |  |  |
| Less than 1 minimum wage | 16 | 18,82 |  | 1,00 | - | | 2 | 2,35 |  | 1,00 | - | 15 | 17,65 |  | 1,00 | - |
| 1 minimum wage or more | 16 | 21,92 |  | 1,16 | 0,63 – 2,17 | | ** | ** |  | ** | ** | 16 | 21,92 |  | 1,24 | 0,66 – 2,34 |
| **Gestational age** |  |  | **0,024** |  |  | |  |  | 0,585^¶^ |  |  |  |  | **0,036** |  |  |
| 1st trimester | ** | ** |  | ** | ** | | ** | ** |  | ** | ** | ** | ** |  | ** | ** |
| 2nt trimester | 10 | 15,15 |  | ** | ** | | ** | ** |  | ** | ** | 10 | 15,15 |  | ** | ** |
| 3rd trimester | 21 | 28,00 |  | ** | ** | | 2 | 2,67 |  | ** | ** | 20 | 26,67 |  | ** | ** |
| **Physical activity** |  |  | **0,298** |  |  | |  |  | 0,617^¶^ |  |  |  |  | 0,247 |  |  |
| No | 23 | 18,40 |  | 1,00 | - | | 2 | 1,60 |  | 1,00 | - | 22 | 17,60 |  | 1,00 | - |
| Yes | 9 | 26,47 |  | 1,44 | 0,73 – 2,82 | | ** | ** |  | ** | ** | 9 | 26,47 |  | 1,50 | 0,76 – 2,96 |
| **Alcoholic beverage** |  |  | 0,574^¶^ |  |  | |  |  | 0,890^¶^ |  |  |  |  | 0,554^¶^ |  |  |
| No | 30 | 20,00 |  | 1,00 | - | | 2 | 1,33 |  | 1,00 | - | 29 | 19,33 |  | 1,00 | - |
| Yes | 2 | 22,22 |  | 1,11 | 0,31 – 3,95 | | ** | ** |  | ** | ** | 2 | 22,22 |  | 1,15 | 0,32 – 4,09 |
| **Myoma** |  |  | 0,335^¶^ |  |  | |  |  | 0,808^¶^ |  |  |  |  | 0,359^¶^ |  |  |
| No | 30 | 20,98 |  | 1,00 | - | | 2 | 1,40 |  | 1,00 | - | 29 | 20,28 |  | 1,00 | - |
| Yes | 2 | 12,50 |  | 0,60 | 0,16 – 2,27 | | ** | ** |  | ** | ** | 2 | 12,50 |  | 0,61 | 0,16 – 2,36 |
| **Polycystic ovaries** |  |  | 0,488^¶^ |  |  | |  |  | 0,731^¶^ |  | - |  |  | 0,520^¶^ |  |  |
| No | 28 | 20,59 |  | 1,00 | - | | 2 | 1,47 |  | 1,00 |  | 27 | 19,85 |  | 1,00 | - |
| Yes | 4 | 17,39 |  | 0,84 | 0,33 – 2,19 | | ** | ** |  | ** | ** | 4 | 17,39 |  | 0,88 | 0,34 – 2,28 |
| **Other gynecological changes** |  |  | 0,492^¶^ |  |  | |  |  | 0,843^¶^ |  |  |  |  | 0,516^¶^ |  |  |
| No | 30 | 20,55 |  | 1,00 | - | | 2 | 1,37 |  | 1,00 | - | 29 | 19,86 |  | 1,00 | - |
| Yes | 2 | 15,38 |  | 0,75 | 0,20 – 2,80 | | ** | ** |  | ** | ** | 2 | 15,38 |  | 0,77 | 0,21 – 2,90 |
| **Had STI** |  |  | 0,354^¶^ |  |  | |  |  | 0,180^¶^ |  |  |  |  | 0,592^¶^ |  |  |
| No | 28 | 19,44 |  | 1,00 | - | | 1 | 0,69 |  | 1,00 | - | 28 | 19,44 |  | 1,00 | - |
| Yes | 4 | 26,67 |  | 1,37 | 0,55 – 3,40 | | 1 | 6,67 |  | 9,60 | 0,63 – 147,03 | 3 | 20,00 |  | 1,03 | 0,35 – 2,99 |
| **STI Treatment** |  |  | 0,409 |  |  | |  |  | 0,169^¶^ |  |  |  |  | 0,848 |  |  |
| No | 4 | 28,57 |  | 1,00 | - | | 1 | 7,14 |  | 1,00 | - | 3 | 21,43 |  | 1,00 | - |
| Yes | 28 | 19,31 |  | 0,68 | 0,28 – 1,65 | | 1 | 0,69 |  | 0,10 | 0,01 – 1,47 | 28 | 19,31 |  | 0,90 | 0,31 – 2,60 |
| **Ectopic pregnancy** |  |  | 0,348^¶^ |  |  | |  |  | 0,926^¶^ |  |  |  |  | 0,332^¶^ |  |  |
| No | 30 | 19,61 |  | 1,00 | - | | 2 | 1,31 |  | 1,00 | - | 29 | 18,95 |  | 1,00 | - |
| Yes | 2 | 33,33 |  | 1,7 | 0,52 – 5,53 | | ** | ** |  | ** | ** | 2 | 33,33 |  | 1,76 | 0,54 – 5,73 |
| **Positive test for STI** |  |  | 0,652^¶^ |  |  | |  |  | 0,926^¶^ |  |  |  |  | 0,668 |  |  |
| No | 31 | 20,26 |  | 1,00 | - | | 2 | 1,31 |  | 1,00 | - | 30 | 19,61 |  | 1,00 | - |
| Yes | 1 | 16,67 |  | 0,82 | 0,13 – 5,09 | | ** | ** |  | ** | ** | 1 | 16,67 |  | 0,85 | 0,14 – 5,26 |
| **Age of first sexual intercourse** |  |  | **0,030** |  |  | |  |  | 0,682^¶^ |  |  |  |  | **0,026**^¶^ |  |  |
| ≽ 15 years | 20 | 16,13 |  | 1,00 | - | | 2 | 1,61 |  | 1,00 | - | 19 | 15,32 |  | 1,00 | - |
| Up to 14 years | 9 | 34,62 |  | 2,15 | 1,10 – 4,18 | | ** | ** |  | ** | ** | 9 | 34,62 |  | 2,26 | 1,15 – 4,43 |
| **Had more than 1 partner during life** |  |  | 0,580 |  |  | |  |  | 0,490^¶^ |  |  |  |  | 0,669 |  |  |
| No | 8 | 17,39 |  | 1,00 | - | | ** | ** |  | ** | ** | 8 | 17,39 |  | 1,00 | - |
| Yes | 23 | 21,30 |  | 1,22 | 0,59 – 2,54 | | 2 | 1,85 |  | - | - | 22 | 20,37 |  | 1,17 | 0,56 – 2,44 |
| **Pain during sexual intercourse** |  |  | 0,835 |  |  | |  |  | 0,670^¶^ |  |  |  |  | 0,759 |  |  |
| No | 25 | 19,69 |  | 1,00 | - | | 2 | 1,57 |  | 1,00 | - | 24 | 18,90 |  | 1,00 | - |
| Yes | 6 | 21,43 |  | 1,09 | 0,49 – 2,41 | | ** | ** |  | ** | ** | 6 | 21,43 |  | 1,13 | 0,51 – 2,52 |
| **Bleeding during sexual intercourse** |  |  | 0,575^¶^ |  |  | |  |  | 0,911^¶^ |  |  |  |  | 0,593^¶^ |  |  |
| No | 30 | 20,27 |  | 1,00 | - | | 2 | 1,35 |  | 1,00 | - | 29 | 19,59 |  | 1,00 | - |
| Yes | 1 | 14,29 |  | 0,70 | 0,11 – 4,48 | | ** | ** |  | ** | ** | 1 | 14,29 |  | 0,73 | 0,11 – 4,64 |
| **Condom use** |  |  | 0,120 |  |  | |  |  | 0,608^¶^ |  |  |  |  | 0,093 |  |  |
| No / Sometimes | 21 | 17,36 |  | 1,00 | - | | 2 | 1,65 |  | 1,00 | - | 20 | 16,53 |  | 1,00 | - |
| Yes | 10 | 29,41 |  | 1,70 | 0,88 -3,25 | | ** | ** |  | ** | ** | 10 | 29,41 |  | 1,78 | 0,92 – 3,44 |
| **Time of last gynecological examination** |  |  | 0,549 |  |  | |  |  | 0,702^¶^ |  |  |  |  | 0,430 |  |  |
| In the last year | 16 | 18,39 |  | 1,00 | - | | 1 | 1,15 |  | 1,00 | - | 15 | 17,24 |  | 1,00 | - |
| More than 1 year | 16 | 22,22 |  | 1,20 | 0,65 – 2,25 | | 1 | 1,39 |  | 1,21 | 0,08 – 19,15 | 16 | 22,22 |  | 1,29 | 0,68 – 2,43 |
| **Use of contraceptives** |  |  | 0,605 |  |  | |  |  | 0,742 |  |  |  |  | 0,471 |  |  |
| No | 17 | 22,08 |  | 1,00 | - | | 1 | 1,30 |  | 1,00 | - | 17 | 22,08 |  | 1,00 | - |
| Yes | 15 | 18,75 |  | 0,85 | 0,46 – 1,58 | | 1 | 1,25 |  | 0,96 | 0,06 – 15,25 | 14 | 17,50 |  | 0,79 | 0,42 – 1,50 |
| **Number of pregnancies** |  |  | 0,444 |  |  | |  |  | 1,000* |  |  |  |  | 0,322 |  |  |
| 1 | 13 | 25,49 |  | 1,00 | - | | 1 | 1,96 |  | 1,00 | - | 13 | 25,49 |  | 1,00 | - |
| 2 | 9 | 20,00 |  | 0,78 | 0,37 – 1,66 | | ** | ** |  | ** | ** | 9 | 20,00 |  | 0,78 | 0,37 – 1,66 |
| 3 or more | 10 | 15,87 |  | 0,62 | 0,30 – 1,30 | | 1 | 1,59 |  | 0,81 | 0,05 – 12,74 | 9 | 14,29 |  | 0,56 | 0,26 – 1,21 |
| **Abortion** |  |  | 0,766 |  |  | |  |  | 0,499^¶^ |  |  |  |  | 0,991 |  |  |
| None | 22 | 19,64 |  | 1,00 | - | | 1 | 0,89 |  | 1,00 | - | 22 | 19,64 |  | 1,00 | - |
| 1 or more | 10 | 21,74 |  | 1,11 | 0,57 – 2,15 | | 1 | 2,17 |  | 2,43 | 0,15 – 38,44 | 9 | 19,57 |  | 0,99 | 0,49 – 2,00 |
| **Premature birth** |  |  | 0,295 |  |  | |  |  | 0,345^¶^ |  |  |  |  | 0,140 |  |  |
| None | 28 | 21,88 |  | 1,00 | - | | 1 | 0,78 |  | 1,00 | - | 28 | 21,88 |  | 1,00 | - |
| 1 or more | 4 | 13,33 |  | 0,61 | 0,23 – 1,61 | | 1 | 3,33 |  | 4,27 | 0,27 – 66,87 | 3 | 10,00 |  | 0,46 | 0,15 – 1,41 |
| **Stillborn** |  |  | 0,541 |  |  | |  |  | 0,834^¶^ |  |  |  |  | 0,541 |  |  |
| None | 27 | 20,61 |  | 1,00 | - | | 1 | 0,76 |  | 1,00 | - | 27 | 20,61 |  | 1,00 | - |
| 1 or more | 4 | 15,38 |  | 0,75 | 0,28 – 1,96 | | ** | ** |  | ** | ** | 4 | 15,38 |  | 0,75 | 0,28 – 1,96 |
| **Expected prenatal consultations** |  |  | 0,323 |  |  | |  |  | 0,584^¶^ |  |  |  |  | 0,375 |  |  |
| Greater than or equal to 6 | 25 | 21,37 |  | 1,00 | - | | 2 | 1,71 |  | 1,00 | - | 24 | 20,51 |  | 1,00 | - |
| Less than 6 | 5 | 13,89 |  | 0,65 | 0,27 – 1,58 | | ** | ** |  | ** | ** | 5 | 13,89 |  | 0,68 | 0,28 – 1,65 |
| **Antibiotic use in the last 3 months** |  |  | 0,146 |  |  | |  |  | 0,590^¶^ |  |  |  |  | 0,105 |  |  |
| No | 17 | 16,67 |  | 1,00 | - | | 1 | 0,98 |  | 1,00 | - | 16 | 15,69 |  | 1,00 | - |
| Yes | 15 | 26,32 |  | 1,58 | 0,85 – 2,92 | | 1 | 1,75 |  | 1,79 | 0,11 – 28,32 | 15 | 26,32 |  | 1,68 | 0,90 – 3,14 |
| **Presence of symptoms** |  |  | 0,909 |  |  | |  |  | 0,447^¶^ |  |  |  |  | 0,949 |  |  |
| None | 5 | 17,24 |  | 1,00 | - | | 1 | 3,45 |  | 1,00 | - | 5 | 17,24 |  | 1,00 | - |
| 1 | 11 | 20,00 |  | 1,16 | 0,44 – 3,03 | | ** | ** |  | ** | ** | 11 | 20,00 |  | 1,16 | 0,44 – 3,03 |
| 2 or more | 16 | 21,05 |  | 1,22 | 0,49 – 3,04 | | 1 | 1,32 |  | 0,38 | 0,02 – 5,95 | 15 | 19,74 |  | 1,14 | 0,46 – 2,87 |
| **Vaginal discharge** |  |  | 0,780 |  |  | |  |  | 0,729^¶^ |  |  |  |  | 0,636 |  |  |
| No | 16 | 21,05 |  | 1,00 | - | | 1 | 1,32 |  | 1,00 | - | 16 | 21,05 |  | 1,00 | - |
| Yes | 16 | 19,28 |  | 0,92 | 0,49 – 1,70 | | 1 | 1,20 |  | 0,92 | 0,06 – 14,51 | 15 | 18,07 |  | 0,86 | 0,46 – 1,62 |
| **Itching** |  |  | 0,512^¶^ |  |  | |  |  | 0,269^¶^ |  |  |  |  | 0,520^¶^ |  |  |
| No | 27 | 19,85 |  | 1,00 | - | | 1 | 0,74 |  | 1,00 | - | 27 | 19,85 |  | 1,00 | - |
| Yes | 5 | 21,74 |  | 1,10 | 0,47 – 2,56 | | 1 | 4,35 |  | 5,91 | 0,38 – 92,03 | 4 | 17,39 |  | 0,88 | 0,34 – 2,28 |
| **Vaginal erythema** |  |  | 0,736^¶^ |  |  | |  |  | 0,938^¶^ |  |  |  |  | 0,667^¶^ |  |  |
| No | 31 | 20,13 |  | 1,00 | - | | 2 | 1,30 |  | 1,00 | - | 30 | 19,48 |  | 1,00 | - |
| Yes | 1 | 20,00 |  | 0,99 | 0,17 – 5,93 | | ** | ** |  | ** | ** | 1 | 20,00 |  | 1,03 | 0,17 – 6,14 |
| **Pelvic pain** |  |  | 0,970 |  |  | |  |  | 0,278^¶^ |  |  |  |  | 0,880 |  |  |
| No | 17 | 20,24 |  | 1,00 | - | | 2 | 2,38 |  | 1,00 | - | 16 | 19,05 |  | 1,00 | - |
| Yes | 15 | 20,00 |  | 0,99 | 0,53 – 1,84 | | ** | ** |  | ** | ** | 15 | 20,0 |  | 1,05 | 0,56 – 1,98 |
| **Genital vesicles** |  |  | 0,507^¶^ |  |  | |  |  | 0,963^¶^ |  |  |  |  | 0,519^¶^ |  |  |
| No | 32 | 20,51 |  | 1,00 | - | | 2 | 1,28 |  | 1,00 | - | 31 | 19,87 |  | 1,00 | - |
| Yes | ** | ** |  | ** | ** | | ** | ** |  | ** | ** | ** | ** |  | ** | ** |
| **Pain when urinating** |  |  | 0,682 |  |  | |  |  | 0,699^¶^ |  |  |  |  | 0,614 |  |  |
| No | 26 | 19,55 |  | 1,00 | - | | 2 | 1,50 |  | 1,00 | - | 25 | 18,80 |  | 1,00 | - |
| Yes | 6 | 23,08 |  | 1,18 | 0,54 – 2,59 | | ** | ** |  | ** | ** | 6 | 23,08 |  | 1,23 | 0,56 – 2,70 |
| **Swollen glands in the groin** |  |  | 0,258^¶^ |  |  | |  |  | 0,110^¶^ |  |  |  |  | 0,550^¶^ |  |  |
| No | 29 | 19,21 |  | 1,00 | - | | 1 | 0,66 |  | 1,00 | - | 29 | 19,21 |  | 1,00 | - |
| Yes | 3 | 33,33 |  | 1,74 | 0,65 – 4,64 | | 1 | 11,11 |  | 16,78 | 1,13 – 249,00 | 2 | 22,22 |  | 1,16 | 0,33 – 4,12 |
| **Other gynecological changes** |  |  | 0,633 |  |  | |  |  | 0,609^¶^ |  |  |  |  | 0,555 |  |  |
| No | 24 | 19,20 |  | 1,00 | - | | 2 | 1,60 |  | 1,00 | - | 23 | 18,40 |  | 1,00 | - |
| Yes | 8 | 22,86 |  | 1,19 | 0,59 – 2,42 | | ** | ** |  | ** | ** | 8 | 22,86 |  | 1,24 | 0,61 – 2,54 |
| **Partner treatment for infertility** |  |  | 0,495^¶^ |  |  | |  |  | 0,787^¶^ |  |  |  |  | 0,524^¶^ |  |  |
| No | 29 | 20,42 |  | 1,00 | - | | 2 | 1,41 |  | 1,00 | - | 28 | 19,72 |  | 1,00 | - |
| Yes | 3 | 16,67 |  | 0,82 | 0,28 – 2,42 | | ** | ** |  | ** | ** | 3 | 16,67 |  | 0,85 | 0,28 – 2,51 |
| **Partner treatment for STIs** |  |  | 0,083^¶^ |  |  | |  |  | 0,889^¶^ |  |  |  |  | 0,075^¶^ |  |  |
| No | 28 | 18,79 |  | 1,00 | - | | 2 | 1,34 |  | 1,00 | - | 27 | 18,12 |  | 1,00 | - |
| Yes | 4 | 44,44 |  | 2,37 | 1,06 – 5,30 | | ** | ** |  | ** | ** | 4 | 44,44 |  | 2,45 | 1,09 – 5,51 |

*n = Absolute frequency; †P = Prevalence; ‡PR = Crude prevalence ratio; §CI95%: Confidence interval 95%; ¶: Fisher's exact test; **Statistical analysis was not possible
